# Sperm proteins SOF1, TMEM95, and SPACA6 are required for sperm−oocyte fusion in mice

**DOI:** 10.1073/pnas.1922650117

**Published:** 2020-05-11

**Authors:** Taichi Noda, Yonggang Lu, Yoshitaka Fujihara, Seiya Oura, Takayuki Koyano, Sumire Kobayashi, Martin M. Matzuk, Masahito Ikawa

**Affiliations:** ^a^Research Institute for Microbial Diseases, Osaka University, 565-0871 Osaka, Japan;; ^b^Graduate School of Pharmaceutical Sciences, Osaka University, 565-0871 Osaka, Japan;; ^c^Division of Molecular Genetics, Shigei Medical Research Institute, 701-0202 Okayama, Japan;; ^d^Center for Drug Discovery, Baylor College of Medicine, Houston, TX 77030;; ^e^Department of Pathology & Immunology, Baylor College of Medicine, Houston, TX 77030;; ^f^The Institute of Medical Science, The University of Tokyo, 108-8639 Tokyo, Japan

**Keywords:** infertility, IZUMO1, sperm−oocyte fusion, LLCFC1, transgenic

## Abstract

Sperm−oocyte membrane fusion is one of the most important events for fertilization. So far, IZUMO1 and Fertilization Influencing Membrane Protein (FIMP) on the sperm membrane and CD9 and JUNO (IZUMO1R/FOLR4) on the oocyte membrane have been identified as fusion-required proteins. However, the molecular mechanisms for sperm−oocyte fusion are still unclear. Here, we show that testis-enriched genes, sperm−oocyte fusion required 1 (*Sof1*/*Llcfc1*/*1700034O15Rik*), transmembrane protein 95 (*Tmem95*), and sperm acrosome associated 6 (*Spaca6*), encode sperm proteins required for sperm−oocyte fusion in mice. These knockout (KO) spermatozoa carry IZUMO1 but cannot fuse with the oocyte plasma membrane, leading to male sterility. Transgenic mice which expressed mouse *Sof1*, *Tmem95,* and *Spaca6* rescued the sterility of *Sof1*, *Tmem95*, and *Spaca6* KO males, respectively. SOF1 and SPACA6 remain in acrosome-reacted spermatozoa, and SPACA6 translocates to the equatorial segment of these spermatozoa. The coexpression of SOF1, TMEM95, and SPACA6 in IZUMO1-expressing cultured cells did not enhance their ability to adhere to the oocyte membrane or allow them to fuse with oocytes. SOF1, TMEM95, and SPACA6 may function cooperatively with IZUMO1 and/or unknown fusogens in sperm−oocyte fusion.

## Significance

The sperm−oocyte fusion step is important to transport the male genome into oocytes. So far, IZUMO1 and FIMP have been identified as fusion-related proteins in spermatozoa, but the molecular mechanisms underpinning sperm−oocyte fusion and all of the proteins required for this essential process remain unclear. In this study, using CRISPR-Cas9−mediated gene knockouts in mice, we discover that sperm proteins SOF1, TMEM95, and SPACA6 are required for sperm−oocyte fusion and male fertility. As these genes are conserved among mammals including human, they may explain not only the sperm−oocyte fusion process but also idiopathic male infertility and be unique targets for contraception.

There are many steps to complete fertilization, such as sperm migration through the female reproductive tract, the physiological and morphological changes of spermatozoa (capacitation, hyperactivation, and acrosome reaction), sperm−oocyte interaction/fusion, and egg activation (1). During the last few decades, gene knockout (KO) studies have revealed many critical proteins for these steps (2–4). However, for the sperm−oocyte fusion step, only four factors have been identified: CD9 and JUNO expressed in oocytes and IZUMO1 and FIMP expressed in spermatozoa.

In 2000, CD9, an integral membrane protein, was unintentionally found to be critical. *Cd9* is ubiquitously expressed in multiple tissues, and *Cd9* KO male and female mice are healthy, but the female mice show severe subfertility due to a sperm−oocyte fusion defect (5–7). The later studies showed that CD9 acts as a scaffolding protein and is required for the normal microvillar shape and distribution (8). In 2005, we reported that IZUMO1 is an essential factor for sperm−oocyte fusion (9). IZUMO1 belongs to the Ig superfamily and is specifically expressed in the testes. IZUMO1 is localized on the inner and outer acrosome membranes in acrosome-intact spermatozoa, and then translocated to the sperm plasma membrane after the acrosome reaction (10, 11). *Izumo1* KO spermatozoa can penetrate the zona pellucida (ZP), but spermatozoa accumulate in the perivitelline space due to a fusion defect (9). Since there is no interaction found between IZUMO1 and CD9, the identification of the IZUMO1 receptor had been challenging. In 2014, by using oligomerized IZUMO1 ectodomains, Bianchi et al. (12) succeeded in identifying a glycosylphosphatidylinositol-anchored protein, JUNO (also known as IZUMO1 receptor [IZUMO1R] and folate receptor 4 [FOLR4]) as an IZUMO1 receptor on the oocyte plasma membrane. IZUMO1 and JUNO form a 1:1 complex (12), and critical residues to form this interaction were identified by X-ray crystal structure analysis (13–15). However, in vitro studies implied that IZUMO1 may be responsible for sperm−oocyte membrane adhesion instead of fusion (16, 17). Therefore, key mechanisms underlying the fusion step are yet to be unveiled.

The emergence of the CRISPR-Cas9 system opened a new era in mammalian genome editing (18–21). The CRISPR-KO approach efficiently screens male fertility genes in vivo. In fact, we revealed that more than 90 genes are dispensable (22–26), while 10 genes and two clusters are required (21, 27–30) for male fertility. Recently, we reported that a testis-specific gene, *4930451I11Rik* (renamed as Fertilization Influencing Membrane Protein [*Fimp*]), is another sperm factor critical for sperm−oocyte fusion (31). While the amount and localization of IZUMO1 in *Fimp* KO spermatozoa are normal, *Fimp* KO males are severely subfertile due to a sperm−oocyte fusion defect. There are two isoforms of FIMP (secreted and transmembrane forms), and we showed that the transmembrane isoform is required for sperm−oocyte fusion using transgenic (Tg) rescued *Fimp* KO males. Whereas IZUMO1-expressing HEK293T cells bound to oolemma, FIMP did not directly mediate HEK293T cells−oolemma binding/fusion or modulate IZUMO1-mediated binding/fusion. Thus, the function of FIMP remains to be studied in more detail.

In the present study, we focused on three testis-enriched genes (sperm−oocyte fusion required 1 [*Sof1*], transmembrane protein 95 [*Tmem95*], and sperm acrosome membrane-associated protein 6 [*Spaca6*]), which are predicted as membrane proteins by in silico analyses. The genes are also conserved in human. *Sof1* is originally registered as *1700034O15Rik* in Mouse Genome Informatics and recently changed to *Llcfc1* because it conserves an “LLLL and CFNLAS” motif, but its physiological function remains to be studied. Thus, we renamed it *Sof1* to represent its physiological function. Bull TMEM95 is localized to the acrosome region, equatorial segment, and connecting piece of spermatozoa (32). Spermatozoa from a bull bearing biallelic nonsense mutation in *TMEM95* are defective in fusion to oocytes (33). These results suggest that TMEM95 is required for the sperm−oocyte interaction, but there remains a chance that unidentified mutations are responsible for infertility. SPACA6 belongs to the Ig superfamily. Lorenzetti et al. (34) reported that *Spaca6* messenger RNA (mRNA) was disrupted in BART97b Tg male mice, which exhibited infertility and a sperm−oocyte fusion defect. It suggests that SPACA6 is critical for sperm−oocyte fusion, but the Tg insertion may affect the expression levels of neighboring genes, rendering the essentiality of SPACA6 inconclusive.

In the present study, we generated *Sof1*, *Tmem95*, and *Spaca6* KO mice using CRISPR-Cas9 and revealed that *Sof1*, *Tmem95*, and *Spaca6* KO males are sterile due to impaired sperm−oocyte fusion. We could not observe any overt defects in the amount and localization of IZUMO1 in the spermatozoa of each mutant mouse. Our results suggest that sperm−oocyte fusion is a complicated process that is mediated by multiple factors. Elucidation of these proteins and their mechanisms of action will propel progress toward our understanding of sperm−oocyte fusion and idiopathic male infertility.

## Results

### In Silico Expression Analyses of Fusion-Related Sperm Genes.

Using published single-cell RNA-sequencing (scRNA-seq) data generated from mouse and human spermatogenic cells at different stages (35), we found that the sperm-expressed proteins known to be involved in sperm−oocyte membrane fusion (e.g., IZUMO1 and FIMP) exhibit an elevated mRNA expression in round spermatids (*SI Appendix*, Fig. S1), the cell type in which the proacrosomal vacuole forms. Specifically, during human and mouse spermatogenesis, these mRNAs are initially detected in early round spermatids, dramatically increase at the mid-spermatid stage, and return to a low level in late round spermatids. Among the genes showing similar expression pattern in silico, and given the possibility that the molecular mechanisms underpinning gamete fusion are evolutionarily conserved, we preferentially focused on sequences that encoded open reading frames (ORFs) in a broad spectrum of species, including but not limited to human and mice. Herein, we concentrated on the functional analyses of *Sof1*, *Tmem95*, and *Spaca6*.

### *Sof1* Gene Deletion Male Mice Are Sterile.

**Fig. 1.**
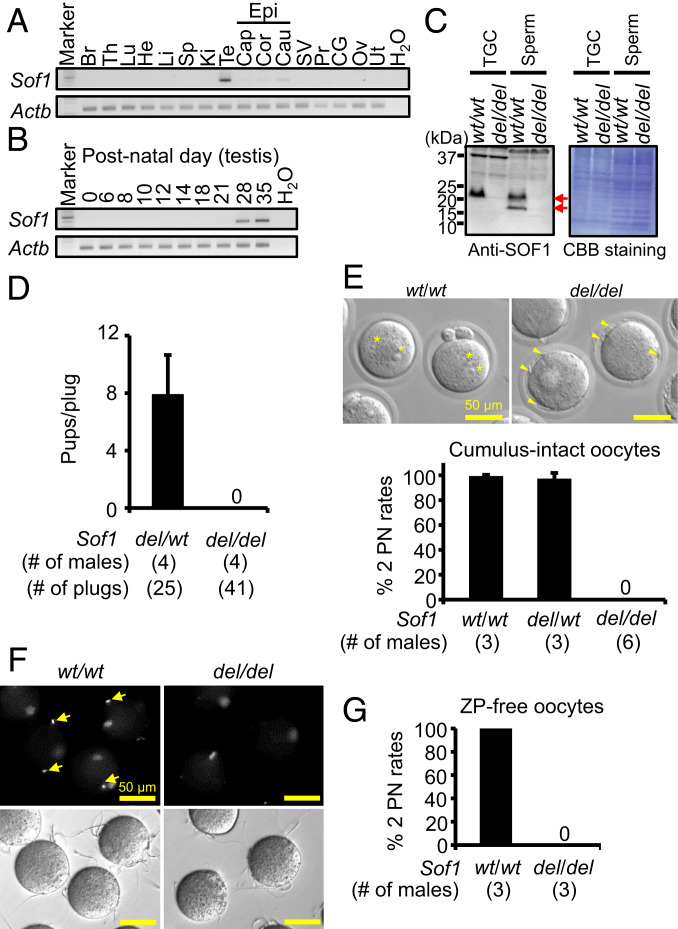
Fertility of *Sof1 del/del* mice. (*A*) Multitissue gene expression by RT-PCR analysis. Mouse *Sof1* is abundantly expressed in a testis. Beta actin (*Actb*) was used as the control. Br, brain; Th, thymus; Lu, lung; He, heart; Li, liver; Sp, spleen; Ki, kidney; Te, testis; Epi, epididymis; Cap, caput epididymis; Cor, corpus epididymis; Cau, cauda epididymis; SV, seminal vesicle; Pr, prostate; Ov, ovary; Ut, uterus. (*B*) Detection of *Sof1* mRNA in testes postnatally. Mouse *Sof1* was detected in testis after postnatal day 28. (*C*) Detection of SOF1 in TGC and spermatozoa. TGC and spermatozoa of wild-type (*wt*) and *Sof1* whole deletion (*del*) males (*SI Appendix*, Fig. S2) were used for Western blot analysis. Specific bands were detected in WT TGC and spermatozoa (red arrows). (*D*) Male fecundity. Each male was caged with two *wt/wt* females for 2 mo. *Sof1 del/del* males succeeded in mating, but females did not deliver any offspring (pups/plug: 7.9 ± 2.8 [*del/wt*], 0 [*del/del*]). The litter sizes of *Sof1 del/wt* and *del/del* males were 9.6 ± 1.2 and 0, respectively. (*E*) Sperm fertilizing ability with cumulus-intact oocytes in vitro. Fertilized eggs with two PN (2 PN) (asterisks) were observed in *Sof1* control spermatozoa (*Upper*). However, *Sof1* KO spermatozoa were accumulated in the perivitelline space (arrowheads, *Upper*), and there were no oocytes bearing 2 PN (*Lower*, fertilization rates: 99.5 ± 0.9% [*wt/wt*, 153 oocytes], 97.3 ± 4.6% [*del/wt*, 159 oocytes], 0% [*del/del*, 375 oocytes]) (Movie S3). (*F*) Sperm−oocyte fusion assay. Spermatozoa were incubated with the ZP-free oocytes prestained with Hoechst 33342 for 30 min. *Sof1* control spermatozoa were stained by Hoechst 33342, indicating that they fused with oocytes (arrows). *Sof1* KO spermatozoa bound to the oolemma, but Hoechst 33342-positive spermatozoa were rarely observed in the *Sof1* KO group (Hoechst 33342-positive spermatozoa/oocyte: 1.12 ± 0.36 [*wt/wt*, *n* = 5, 110 oocytes], 0.06 ± 0.07 [*del/del*, *n* = 5, 119 oocytes], *P* < 0.01). Two mutant lines (d1024 and d1039; *SI Appendix*, Fig. S1) were used for this assay. (*G*) Sperm fertilizing ability with ZP-free oocytes in vitro. *Sof1* KO spermatozoa could not fertilize oocytes (fertilization rates: 100% [*wt/wt*, *n* = 3, 50 oocytes], 0% [*del/del*, *n* = 3, 64 oocytes]).

We performed expression analysis by RT-PCR with multiple tissues, and *Sof1* mRNA is abundantly expressed in the testis (Fig. 1*A* and *SI Appendix*, Table S1). *Sof1* mRNA was detected in testis after postnatal day 28 (Fig. 1*B*), indicating that *Sof1* mRNA is expressed in spermatids. SOF1 is conserved in almost all mammals based on the Ensemble database (accession #ENSMUSG00000029867). The identity of bull, hamster, human, and rat SOF1 compared to mouse SOF1 is 62%, 70%, 49%, and 76%, respectively (*SI Appendix*, Fig. S2*A*). SOF1 contains the conserved sequence motifs “LLLL” and “CFN(L or S)AS” in these species, but the functions of these domains are unclear.

To examine the physiological function of SOF1, we generated *Sof1* mutant mice by introducing a guide RNA (gRNA)/Cas9 expression vector into oocytes (for indel) and embryonic stem (ES) cells (for deletion) (*SI Appendix*, Fig. S2*B* and Table S2). We established three mutant lines carrying 1-base pair (bp) (endonuclease-mediated mutation [*em*] 1), 1,024-bp (*em2*), or 1,039-bp (*em3*) deletions (*SI Appendix*, Fig. S2 *C*–*E*). As mice lacking 1,024 bp and 1,039 bp delete the SOF1 ORF, these mice were used for subsequent analyses. SOF1 is detected in testicular germ cells (TGC) and spermatozoa of wild-type (*wt*) mice, but these bands disappear in *Sof1* gene deletion (*del*)*/del* mice (Fig. 1*C*). We could not obtain fertilized eggs from females mated with *Sof1 del/del* males (*SI Appendix*, Fig. S2*F* and Movies S1 and S2). Further, when *Sof1 del/del* males were caged with *wt/wt* females for 2 mo, the males failed to sire offspring (Fig. 1*D*). In contrast, *Sof1 del/del* females are fertile. There was no difference in the fertilizing ability between *Sof1 del/del* (*em2*) and *Sof1 del/del* (*em3*) males in vivo, and we therefore used *Sof1 del/del* (*em2*) for the subsequent experiments, unless otherwise specified.

### *Sof1* KO Spermatozoa Exhibit Defective Fusion Ability with Oolemma.

**Fig. 2.**
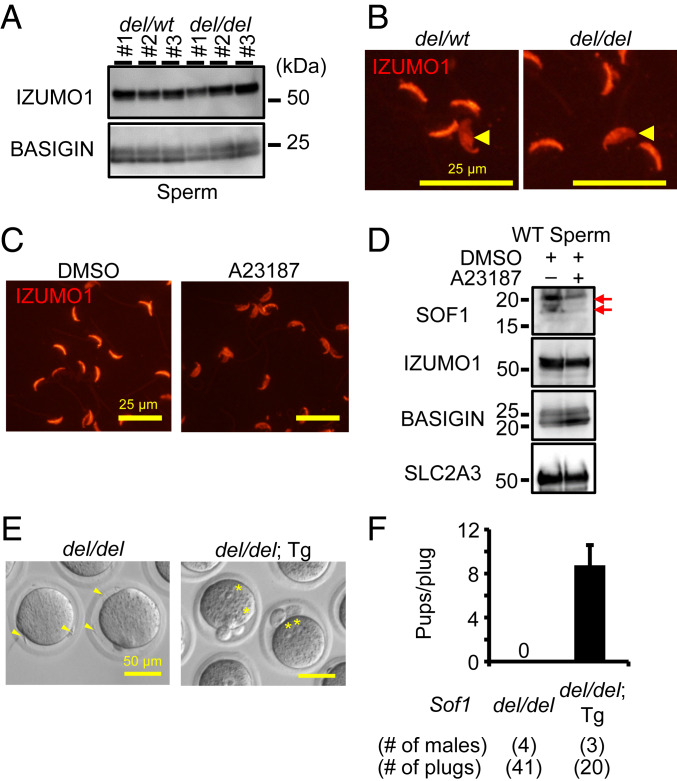
Characterization of *Sof1* KO spermatozoa and Tg rescue. (*A*) Detection of IZUMO1 in spermatozoa. IZUMO1 was detected in *Sof1* control and KO spermatozoa at a comparable level. BASIGIN, a sperm tail protein, was used as a loading control. (*B*) Immunostaining of IZUMO1 in spermatozoa before and after the acrosome reaction. After the acrosome reaction, IZUMO1 in *Sof1* KO spermatozoa spread out over the entire head (arrowheads), indicating that the IZUMO1 localization of *Sof1* KO spermatozoa is normal. (*C*) Inducement of sperm acrosome reaction using Ca^2+^ ionophore A23187. To examine acrosome reaction rates, spermatozoa were stained with an anti-IZUMO1 antibody. DMSO was used as the control. Acrosome reaction rates of spermatozoa treated with DMSO and A23187 were 19.7 ± 10.8% (*n* = 4) and 83.3 ± 5.5% (*n* = 4), respectively. (*D*) Detection of SOF1 in A23187-treated spermatozoa. The doublet bands of SOF1 were detected in spermatozoa treated with DMSO. The lower band disappeared in A23187-treated spermatozoa, but the upper band remained. There was no significant difference in the amount of IZUMO1 between DMSO- and A23187-treated spermatozoa, in agreement to the previous report (10). BASIGIN and SLC2A3, which are mainly localized in sperm tail, were used as the control. (*E*) Oocyte observation of females mated with *Sof1 del/del* males having the *Sof1*-PA Tg insertion. *Sof1* KO spermatozoa were accumulated in the perivitelline space (arrowheads), but spermatozoa of *Sof1*-PA Tg rescued *Sof1 del/del* males (*del/del*; Tg) could fertilize oocytes (asterisks) (2 PN rates: 0% [*del/del*, 109 oocytes, *n* = 3], 100% [*del/del*; Tg, 39 oocytes, *n* = 3]). (*F*) Male fecundity of *Sof1*-PA Tg rescue mice. Each male was caged with two *wt/wt* females for more than 1 mo. The females mated with *Sof1*-PA Tg rescue males could deliver offspring (pups/plug: 0 [*del/del*], 8.7 ± 1.8 [*del/del*; Tg]). The litter sizes of *Sof1 del/del* and *Sof1*-PA Tg rescue males were 0 and 10.2 ± 0.5, respectively. The data of fecundity in *Sof1 del/del* males were reused data of Fig. 1*D*.

To reveal the cause of the male sterility, we first analyzed spermatogenesis of *Sof1* mutant males. There was no difference in the testis morphology and weights between *Sof1 del/wt* and *del/del* males (*SI Appendix*, Fig. S3 *A* and *B*). We could not observe any overt defects in spermatogenesis of *Sof1 del/del* males (*SI Appendix*, Fig. S3*C*). The morphology of *Sof1* KO spermatozoa is normal, and the motility parameters of *Sof1* KO spermatozoa are comparable to control spermatozoa, by computer-assisted sperm analysis (CASA) (*SI Appendix*, Fig. S3 *D* and *E*). When *Sof1* mutant spermatozoa were used for in vitro fertilization (IVF) with cumulus-intact oocytes, *Sof1* KO spermatozoa could penetrate the ZP, but they accumulated in the perivitelline space (Fig. 1 *E* and Movie S3). *Sof1* KO spermatozoa could not fertilize oocytes (Fig. 1 *E*). To analyze the cause of sperm accumulation in the perivitelline space, we examined sperm−oocyte fusion ability using ZP-free oocytes. Although *Sof1* KO spermatozoa bound to the oocyte membrane (number of sperm bound/oocyte: 7.6 ± 3.4 [control], 9.0 ± 4.4 [*Sof1 *KO]), we rarely observed fused spermatozoa by staining with Hoechst 33342 (Fig. 1*F*). Consistently, ZP-free oocytes incubated with *Sof1* KO spermatozoa were not fertilized (Fig. 1*G*). Thus, *Sof1* KO spermatozoa cannot fuse with the oocyte membrane, causing the male sterility. To interrogate whether the defective fusion ability was attributed to the disruption of IZUMO1, we examined the amount and localization of IZUMO1 in *Sof1* KO spermatozoa. The signal intensity of IZUMO1 in *Sof1* KO spermatozoa was comparable to control spermatozoa (Fig. 2*A*), and we could not observe overt defects in IZUMO1 localization in *Sof1* KO spermatozoa before and after the acrosome reaction (Fig. 2*B*). We next examined whether SOF1 remains in acrosome-reacted spermatozoa. We incubated spermatozoa in Toyoda–Yokoyama–Hosi (TYH) drops containing Ca^2+^ ionophore A23187 for 1 h to induce acrosome reaction, and these spermatozoa were subsequently subjected to immunostaining. Almost all of spermatozoa were demonstrated to be acrosome-reacted by IZUMO1 staining (Fig. 2*C*). The sperm extracts were probed with an anti-SOF1 antibody. Both forms of SOF1 decreased in A23187-treated spermatozoa, but the upper form remained (Fig. 2*D*); see *Discussion*.

### Sterility of *Sof1 del/del* Male Mice Is Rescued by a Transgene.

To confirm that the *Sof1* disruption is responsible for the phenotype, we generated Tg mice in which mouse SOF1 with a PA tag were expressed using the testis-specific *Clgn* promoter (*SI Appendix*, Fig. S2 *G* and *H*) on the *Sof1 del/del* background. When Tg-positive *Sof1 del/del* males were mated with *wt/wt* females, we could collect two pronuclei (PN; fertilized) eggs and obtain offspring (Fig. 2 *E* and *F*). Thus, the *Clgn*-*Sof1* transgene rescued the defect and confirms that SOF1 is required for sperm−oocyte fusion and male fertility.

### *Tmem95 del/del* Males Are Sterile.

**Fig. 3.**
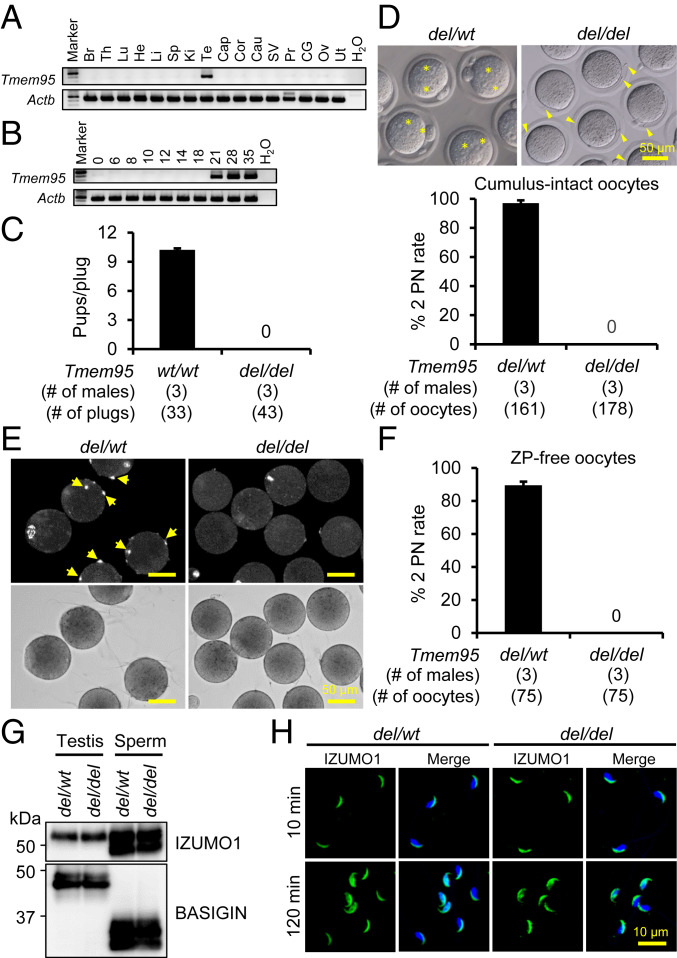
Analyses of *Tmem95 del/del* male mice. (*A*) Detection of *Tmem95* mRNA in mouse tissues and organs by RT-PCR. (*B*) Detection of *Tmem95* mRNA in mouse testes postnatally. (*C*) Analysis of the fecundity of *Tmem95 del/del* males by mating tests. Males were caged with *wt/wt* females for 10 wk, during which copulation plugs were examined and litter sizes were recorded. The *wt/wt* males were examined in the meantime as a positive control. During the mating test, while the *wt/wt* mating pairs delivered 337 offspring with an average litter size of 10.2 ± 1.9, the *del/del* males sire no pup. (*D*) IVF analysis of the ability of *Tmem95* KO spermatozoa to fertilize cumulus-intact oocytes. (*Upper*) After 6 h of incubation, *Tmem95* control spermatozoa successfully inseminated oocytes indicated by the formation of 2 PN (asterisks), whereas the KO spermatozoa were accumulated in the perivitelline space and cannot fertilize the oocytes (arrowheads). (*Lower*) The control spermatozoa give a high fertilization rate of about 97%. In contrast, no 2 PN eggs were observed when inseminated with the KO spermatozoa. (*E*) Analysis of the ability of *Tmem95* KO spermatozoa to fuse with the oocyte plasma membrane. Spermatozoa were incubated with Hoechst 33342-preloaded oocytes for 30 min, and the membrane fusion was determined by the transfer of Hoechst 33342 fluorescence signal to fused sperm heads (yellow arrows). The lower bright-field images indicate both *Tmem95* control and KO spermatozoa could bind normally to the oocyte surface. (*F*) IVF analysis of the ability of *Tmem95* KO spermatozoa to fuse with ZP-free oocytes. While about 97% of the *Tmem95* control sperm-inseminated oocytes formed 2 PN, the KO spermatozoa could not fuse with ZP-free oocytes at all. (*G*) Expression of IZUMO1 in *Tmem95* KO spermatozoa. The expression of IZUMO1 was comparable in the testis and sperm lysates of *Tmem95 del/wt* and *del/del* males. The expression of BASIGIN was analyzed in parallel by immunoblotting as a loading control. (*H*) Localization of IZUMO1 in *Tmem95* KO spermatozoa before and after the acrosome reaction. After 10 and 120 min of incubation in TYH medium, the localization of IZUMO1 in the control and KO spermatozoa was visualized by immunocytochemistry. Sperm nuclei were labeled by Hoechst 33342. Same as control sperm, IZUMO1 in the KO spermatozoa was localized to the acrosome cap before the acrosome reaction and translocated to the equatorial segment or the entire sperm head after the acrosome reaction.

As depicted by RT-PCR analyses (using primers as shown in *SI Appendix*, Table S1), *Tmem95* was exclusively expressed in mouse testis, and testis expression was first observed on day 21 postpartum, during which spermiogenesis initiates (Fig. 3 *A* and *B*). Such a pattern of postnatal testis expression is in agreement with the in silico analysis using the aforementioned scRNA-seq data, in which the mRNA expression peaks at the mid-spermatid stage in human but is below the level of detection in mouse (*SI Appendix*, Fig. S1). *Tmem95* is widely conserved, and its protein sequences show a high similarity among mammalian species, such as bull, hamster, human, mouse, and rat (*SI Appendix*, Fig. S4*A*). To generate *Tmem95* KO mice, two gRNAs were designed to target the 5′ region upstream of the first exon and 3′ untranslated region (UTR) of *Tmem95* locus (*SI Appendix*, Fig. S4*B*). The mouse line was generated by introducing CRISPR RNA (crRNA)/*trans*-activating crRNA/Cas9 ribonucleoprotein complexes into two PN mouse zygotes via electroporation. *Tmem95 wt/wt*, *del/wt*, and *del/del* mice were distinguished by genomic PCR using forward and reverse primers targeting the 5′ and 3′ regions of *Tmem95*, respectively (*SI Appendix*, Fig. S4 *B* and *C* and Table S2). Sanger sequencing of the KO allele indicated that the mutant mice carry a 1,919-bp deletion in the *Tmem95* locus (*SI Appendix*, Fig. S4*D*). By pairing male mice consecutively with *wt/wt* females for 10 wk, we discovered that the *del/del* males fail to sire any offspring, despite an observation of 43 copulation plugs during the mating period. The *wt/wt* pairs that served as a positive control gave an average litter size of 10.2 ± 1.9 (Fig. 3*C*). The fecundity of *del/del* females was unaffected, as demonstrated by normal litter sizes observed from mating between *Tmem95 del/wt* males and *del/del* females. To investigate whether absence of *Tmem95* leads to pathological abnormalities in the male reproductive system, the gross appearance and histological sections of testes and epididymides and the morphology and motility of spermatozoa from *del/del* males were observed in parallel with analyses of tissues and specimens from *del/wt* littermates. However, the testis appearance, size, and weight in *del/del* males are comparable to heterozygotes (*SI Appendix*, Fig. S5 *A* and *B*). No distinct difference or abnormality was observed in the composition, quantity, and morphology of spermatogenic cells in the seminiferous tubules at each stage and spermatozoa in the caput and cauda epididymides (*SI Appendix*, Fig. S5*C*). *Tmem95* KO spermatozoa showed normal morphology (*SI Appendix*, Fig. S5*D*) and normal motility as determined by CASA (*SI Appendix*, Fig. S5 *E* and *F*).

### *Tmem95* KO Spermatozoa Exhibit Impaired Fusion Ability with Oolemma.

Since *Tmem95 del/del* males show normal sperm production and behavior, IVF analysis was conducted to further understand the cause of the male sterility. After 6 h of insemination, KO spermatozoa were unable to fertilize WT oocytes and accumulated in the perivitelline space (Fig. 3 *D*, *Upper* and Movie S4). In comparison, ∼97% of oocytes were fertilized by control spermatozoa, as reflected by formation of two PN (Fig. 3*D*). Because membrane fusion ability of *Tmem95* KO spermatozoa might be compromised by depletion of *Tmem95*, a fusion assay was performed using Hoechst 33342-preloaded ZP-free oocytes. Control spermatozoa could fuse with the plasma membrane of oocytes, as indicated by the transfer of Hoechst 33342 signal to the internalized sperm heads. Although the binding index of *Tmem95* KO spermatozoa to the oocyte surface (8.7 ± 1.3 spermatozoa per oocyte) was comparable to that of control spermatozoa (9.8 ± 1.5 spermatozoa per oocyte), the KO spermatozoa could rarely fuse with the oocyte membrane, as shown (Fig. 3*E*). The average numbers of control and KO spermatozoa that fused to the oocyte membrane were 1.09 ± 0.03 and 0.17 ± 0.02, respectively. To further verify the inability of *Tmem95* KO spermatozoa to fuse with the oocyte plasma membrane, IVF analysis was conducted using ZP-free oocytes. After 6 h of insemination, about 89% of ZP-free oocytes were fertilized by control spermatozoa, whereas no oocytes were found to be fertilized by KO spermatozoa (Fig. 3*F*).

To explore the probability that the fusion defect originated from impaired functioning of IZUMO1, protein amount and localization were investigated by immunoblot analysis and immunocytochemistry, respectively. As shown in Fig. 3*G*, the amount of IZUMO1 protein was equally detected in testis and sperm lysates of *del/wt* and *del/del* males. In addition, the localization of IZUMO1 was examined in both control and KO spermatozoa after 10 and 120 min of incubation in TYH medium. Similar to control spermatozoa, IZUMO1 localized to the acrosome cap in the acrosome-intact KO spermatozoa and relocated to the equatorial segment or the entire sperm head of acrosome-reacted KO spermatozoa (Fig. 3*H*). The translocation of IZUMO1 indicated that IZUMO1 functioned normally in *Tmem95* control and KO spermatozoa, and the spermatozoa could undergo a normal acrosome reaction. Thus, defective fusion ability of *Tmem95* KO spermatozoa was not ascribed to the dislocation or dysfunction of IZUMO1.

### Infertility of *Tmem95 del/del* Mice Is Restored by a Transgene.

To confirm that the impaired sperm fusion ability in *Tmem95* KO spermatozoa originated from the removal of *Tmem95*, Tg mice expressing 1D4-tagged TMEM95 were generated (*SI Appendix*, Fig. S6 *A* and *B*). *Tmem95 del/del* females that had been paired with a *Tmem95 del/del*; Tg male mouse delivered 31 offspring in four litters (7.8 ± 0.5 pups per litter; *SI Appendix*, Fig. S6*C*), indicating that the KO phenotype was rescued by the transgene. When pairing *Tmem95 del/del*; Tg males with hormone-treated *wt*/*wt* females, a high fertilization rate of 94.4% ± 4.4% was observed (*n *= 3). Immunoblot analysis detected expression of the TG protein in both Tg testis and spermatozoa. Localization of TMEM95 could not be determined by immunostaining, probably due to the low level of expression of the TG protein in Tg spermatozoa (yellow arrowhead; *SI Appendix*, Fig. S6*D*).

### *Spaca6 del/del* Male Mice Are Sterile.

**Fig. 4.**
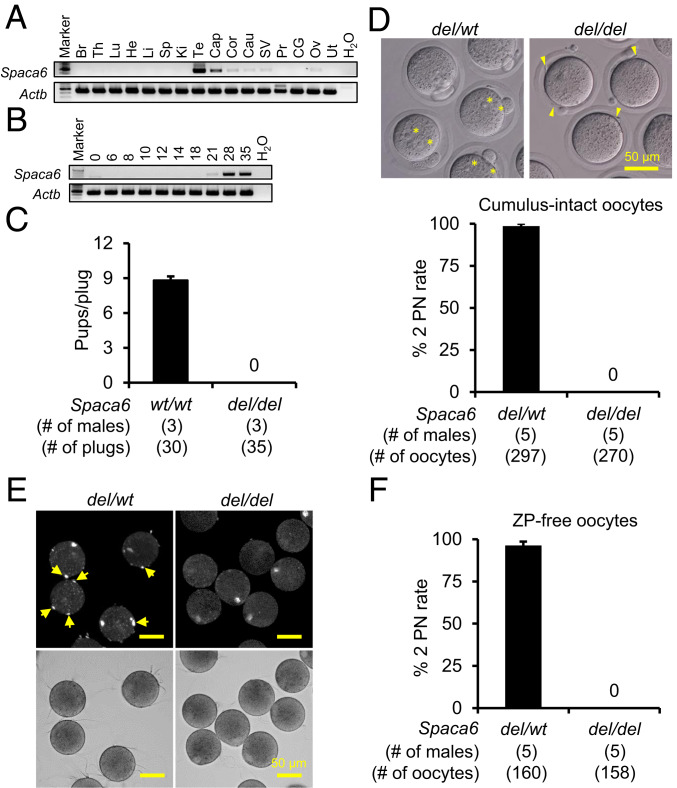
Analyses of *Spaca6 del/del* male mice. (*A*) Detection of *Spaca6* mRNA in various mouse tissues and organs by RT-PCR. (*B*) Detection of *Spaca6* mRNA in mouse testes at various postnatal days by RT-PCR. (*C*) Analysis of the fecundity of *Spaca6 del/del* males by mating tests. Males were caged with *wt/wt* females for 10 wk, during which copulation plugs were examined and litter sizes were recorded. The *wt/wt* males were examined, in the meantime, as a positive control. While the *wt/wt* mating pairs delivered 264 offspring with an average litter size of 8.8 ± 2.4, the *del/del* males failed to sire offspring. (*D*) IVF analysis of the ability of *Spaca6* KO spermatozoa to fertilize cumulus-intact oocytes. (*Upper*) After 6 h of incubation, *Spaca6* control spermatozoa successfully inseminated oocytes indicated by the formation of 2 PN (asterisks), whereas the KO spermatozoa were accumulated in the perivitelline space and cannot fertilize the oocytes (yellow arrowheads). (*Lower*) The control spermatozoa gave a high fertilization rate of about 97%. In contrast, no 2 PN eggs were observed when incubated with KO spermatozoa. (*E*) Analysis of the ability of *Spaca6* KO spermatozoa to fuse with the oocyte plasma membrane. Spermatozoa were incubated with Hoechst 33342-preloaded oocytes for 30 min, and the membrane fusion was determined by transfer of Hoechst 33342 fluorescence signal to fused sperm heads (yellow arrows). The lower bright-field images indicate both control and KO spermatozoa could bind normally to the oocyte surface. (*F*) IVF analysis of the ability of *Spaca6* KO spermatozoa to fuse with ZP-free oocytes. While about 97% of the control sperm-inseminated oocytes formed 2 PN, KO spermatozoa could not fuse with ZP-free oocytes.

*Spaca6* has been previously suggested to be involved in membrane fusion between spermatozoa and oocytes in mice (34). The expression of *Spaca6* mRNA exhibits a strong bias toward the testis, as determined by RT-PCR analysis using complementary DNA (cDNA) extracted from multiple mouse tissues and organs (primers as shown in *SI Appendix*, Table S1). Minor expression was also detected in the epididymis, seminal vesicle, and ovary (Fig. 4*A*). In corroboration with the expression pattern obtained from the scRNA-seq analysis of spermatogenic cells, the expression of *Spaca6* mRNA was initially detected on postnatal day 21, the onset of spermiogenesis (Fig. 4*B*). Similar to other fusion-related sperm proteins, the in silico prediction indicated that *Spaca6* exhibits peak mRNA expression at the mid-round spermatid stage in both humans and mice (*SI Appendix*, Fig. S1).

Distinct from *Sof1* and *Tmem95*, *Spaca6* showed a broader range of evolutionary conservation, according to various public databases; orthologs of SPACA6 have been annotated not only in mammals (e.g., bull, hamster, human, mouse, and rat) but also in nonmammalian vertebrates such as fish (e.g., zebrafish) with less homology (*SI Appendix*, Fig. S7*A*). Based on public databases such as UniProt and Simple Modular Architecture Research Tool (SMART), SPACA6 displays similar domain structures compared with IZUMO1. Both proteins have a short signal peptide of about 20 amino acids (aa) at the N termini, an Ig-like domain of about 85 aa, an *N*-linked glycosylation site upstream of a type I transmembrane domain of 20 aa, and a short cytosolic tail at the C terminus (*SI Appendix*, Fig. S7*B*).

The *Spaca6 del/del* mouse line was generated by zygote electroporation of Cas9 and gRNAs targeting the first coding exon and the region downstream of the 3′ UTR (*SI Appendix*, Fig. S7*C*). The KO and *wt* alleles were identified by genomic PCR using primers targeting the 5′ and 3′ regions of *Spaca6* and primers flanking the first exon, respectively (*SI Appendix*, Fig. S7 *C* and *D* and Table S2). Mutant mice bearing an 8,085-bp deletion and a 4-bp insertion were validated by Sanger sequencing of the mutated locus (*SI Appendix*, Fig. S7*E*). Mating tests indicated that *Spaca6 del/del* males could not sire any offspring when caged with *wt/wt* females for 10 wk, despite producing 35 vaginal plugs during the mating period. In contrast, the average litter size obtained from the *wt/wt* breeding pairs was 8.8 ± 2.4 (Fig. 4*C*). Although there is a trace amount of *Spaca6* mRNA expression in the ovary, depletion of *Spaca6* did not cause any adverse effect on female fecundity, as demonstrated by normal numbers of offspring obtained per litter from pairing *Spaca6 del/wt* males with *del/del* females.

Phenotypic analyses were carried out to investigate whether the infertility of *Spaca6 del/del* males was derived from abnormalities in the male reproductive system. Testis appearance, size, and weight in *del/del* males were normal compared to their *del/wt* littermates (*SI Appendix*, Fig. S8 *A* and *B*). The *del/del* males also showed normal histology of testes and epididymides (*SI Appendix*, Fig. S8*C*), normal sperm morphology (*SI Appendix*, Fig. S8*D*), and normal sperm motility (*SI Appendix*, Fig. S8 *E* and *F*).

### *Spaca6 *KO Spermatozoa Exhibit Impaired Fusion Ability with Oolemma.

The function of *Spaca6* KO spermatozoa was further interrogated by IVF to uncover the cause of the male sterility. After 6 h of incubation in TYH medium, over 98% of the cumulus-intact oocytes were successfully fertilized by *Spaca6* control spermatozoa, as indicated by the presence of two PN. In contrast, no embryos were observed when inseminated with KO spermatozoa (Fig. 4*D*). Similar to the aforementioned *Sof1* and *Tmem95* KO spermatozoa, *Spaca6* KO spermatozoa accumulated in the perivitelline space of WT oocytes (Movie S5), indicating that the KO spermatozoa were able to penetrate the ZP but could not fuse with the oocyte plasma membrane (Fig. 4*D*). Thus, a sperm−oocyte fusion assay was carried out to investigate the ability of *Spaca6* KO spermatozoa to fuse with the oocyte membrane. Similar to control spermatozoa (8.3 ± 1.7 spermatozoa per oocyte), KO spermatozoa could adhere to the oocyte surface with a binding index of 8.4 ± 2.7 spermatozoa per oocyte. However, *Spaca6* KO spermatozoa were rarely found to fuse with the oocyte plasma membrane (Fig. 4*E*). The average numbers of control and KO spermatozoa fused with the oocyte plasma membrane were 1.6 ± 0.13 and 0.07 ± 0.07, respectively. According to the IVF analysis, ∼96% of the WT ZP-free oocytes were fertilized by control spermatozoa, while no fertilized eggs were observed in the *Spaca6* KO sperm-inseminated group (Fig. 4*F*).

**Fig. 5.**
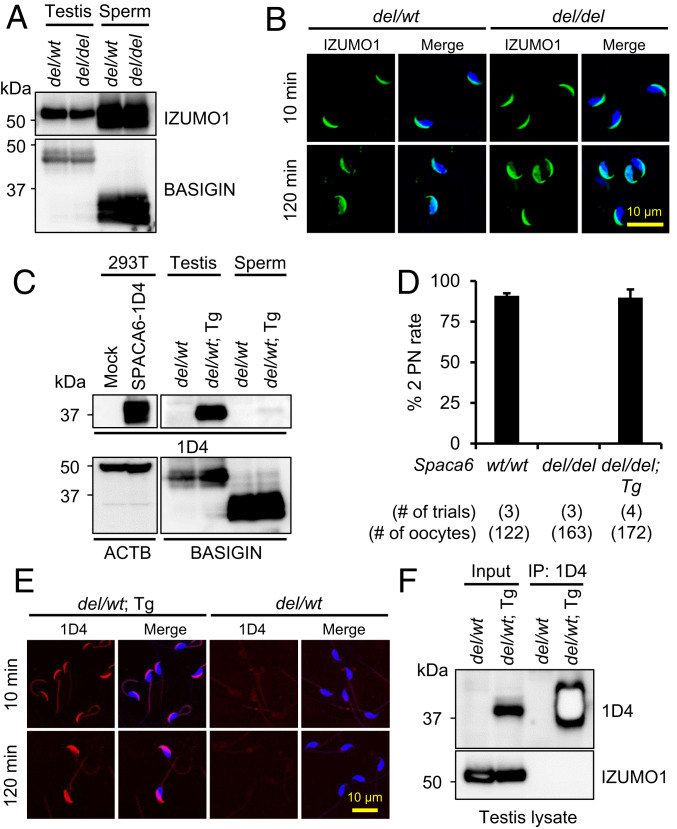
Analyses of *Spaca6*-1D4 Tg male mice. (*A*) Expression of IZUMO1 in *Spaca6* KO spermatozoa. The expression of IZUMO1 was comparable in the testis and sperm lysates of *del/wt* and *del/del* males. The expression of BASIGIN was analyzed in parallel by immunoblot analysis as a loading control. (*B*) Localization of IZUMO1 in *Spaca6* KO spermatozoa before and after the acrosome reaction. After 10 and 120 min of incubation in TYH medium, the localization of IZUMO1 in control and KO spermatozoa was visualized by immunocytochemistry. Sperm nuclei were labeled by Hoechst 33342. Same as control spermatozoa, IZUMO1 in *Spaca6* KO spermatozoa was localized to the acrosome cap before acrosome reaction and translocated to the equatorial segment or the entire sperm head after acrosome reaction. (*C*) Expression of SPACA6-1D4 in testes and cauda epididymal spermatozoa of Tg male mice. Testis and sperm lysates from *del/wt*; Tg males (*del/wt*; Tg) were used to test Tg expression. HEK293T cells expressing SPACA6-1D4 were analyzed as a positive control, and the testis and sperm lysates from *del/wt* males were used as a negative control. The expression of ACTB and BASIGIN was analyzed in parallel as loading controls for the immunoblotting of HEK293T lysates and testis/sperm lysates, respectively. (*D*) Analysis of the fecundity of *Spaca6 del/del*; Tg males expressing SPACA6-1D4. Male mice were paired with hormone-treated *wt/wt* females, and oocytes were harvested from the females about 9 h after copulation. *Spaca6 wt/wt* and *del/del* males were analyzed as positive and negative controls, respectively. The fecundity of two *Spaca6 del/del*; Tg males were tested twice each (for a total of four trials). (*E*) Localization of SPACA6-1D4 in the spermatozoa of Tg mice before and after the acrosome reaction. After 10 and 120 min of incubation in TYH medium, the localization of SPACA6-1D4 in the spermatozoa of *del/wt*; Tg males was visualized by immunocytochemistry. Spermatozoa from *del/wt* males were analyzed in parallel as a negative control. The sperm nuclei were labeled by Hoechst 33342. SPACA6 was localized to the acrosome cap before acrosome reaction and spread out toward the equatorial segment after acrosome reaction. (*F*) Co-IP and Western blot analysis of the interaction between IZUMO1 and SPACA6. Testis lysate from *del/wt*; Tg males was incubated with anti-1D4 antibody-conjugated magnetic beads, and the eluted protein complex was separated by SDS/PAGE and analyzed by Western blot analysis. IZUMO1 was not detected in the co-IP product.

Immunoblotting and immunocytochemistry were further employed to assess the amount and localization of IZUMO1 in *Spaca6* KO spermatozoa. IZUMO1 was equally existed in testis and spermatozoa of *del/wt* and *del/del* males (Fig. 5*A*), and IZUMO1 localization was normal in both acrosome-intact and reacted KO spermatozoa (Fig. 5*B*).

### Sterility of *Spaca6 del/del* Male Mice Is Rescued by a Transgene.

To further characterize the behavior and possible function of SPACA6 during sperm−oocyte fusion, a Tg mouse line expressing 1D4-tagged SPACA6 was generated (*SI Appendix*, Fig. S9 *A* and *B* and Table S3). As shown in Fig. 5*C*, 1D4-tagged SPACA6 was observed in both testis and sperm lysates, whereas no signal was detected in the testis or spermatozoa of *del/wt* males that did not carry the transgene. When *Spaca6 del/del*; Tg males were paired with hormone-treated *wt/wt* females, 90.9% ± 2.7% of the oocytes were fertilized similar to control males (89.7 ± 10.2%; Fig. 5*D* and *SI Appendix*, Fig. S9*C*). In addition, housing of *Spaca6 del/del*; Tg males with WT females for 6 wk resulted in normal litter sizes (8.7 ± 0.1 [*n* = 4 males]; 15 plugs; 15 litters; 130 total offspring). These results indicate that the 1D4-tagged SPACA6 rescued the impaired fusion ability of KO spermatozoa. The localization of 1D4-tagged SPACA6 was subsequently visualized by immunocytochemistry. The tagged SPACA6 was localized to the acrosome cap before the acrosome reaction and translocated to the equatorial segment after the acrosome reaction (Fig. 5*E*). To investigate whether SPACA6 could interact with IZUMO1, coimmunoprecipitation (co-IP) was performed using testis lysates from *del/wt* males with or without transgene. IZUMO1 was not detected in the protein complex precipitated using anti-1D4 antibody-conjugated magnetic beads (Fig. 5*F*), suggesting that IZUMO1 did not form a complex with SPACA6 in the testis.

### SOF1, TMEM95, and SPACA6 Are Conserved in Mammals.

**Fig. 6.**
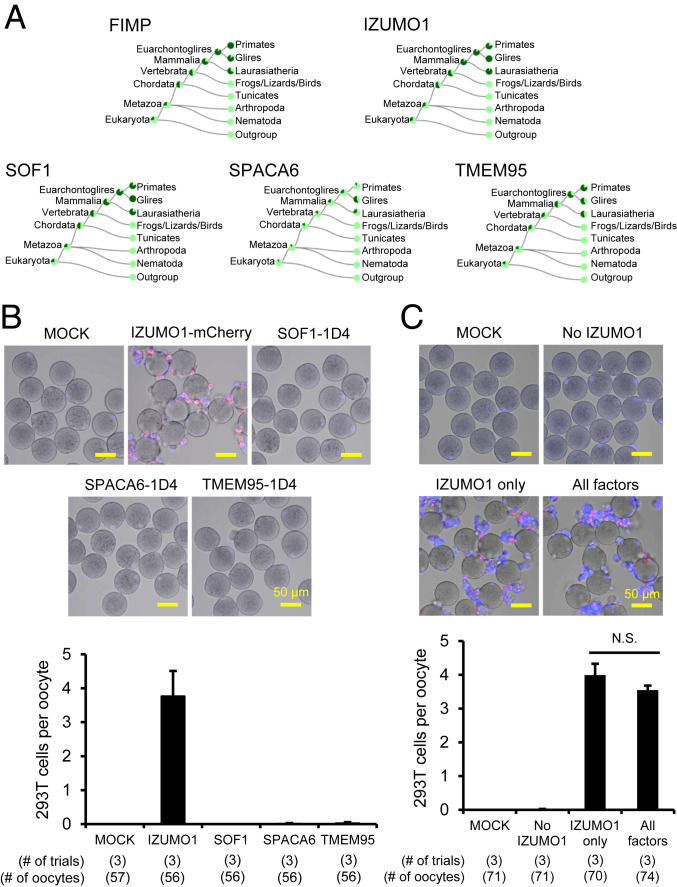
Phylogenetic analyses of the fusion-related sperm proteins and binding assays between ZP-free oocytes and HEK293T cells overexpressing the fusion proteins. (*A*) Phylogenetic trees of FIMP, IZUMO1, SOF1, SPACA6, and TMEM95 using TreeFam. The presence of orthologs is indicated by dark green. Light green indicates loss of an ortholog in several species within a taxon. (*B*) Comparison of oocyte binding ability of HEK293T cells individually expressing recombinant IZUMO1, SOF1, SPACA6, and TMEM95. ZP-free oocytes were incubated with Hoechst 33342-preloaded HEK293T cells overexpressing different fusion-related sperm proteins. Only HEK293T cells expressing IZUMO1-mCherry could adhere to the ZP-free oocytes. (*C*) Comparison of oocyte binding ability of HEK293T cells simultaneously overexpressing various fusion-related sperm factors. HEK293T cells expressing all factors except for IZUMO1 (shown as No IZUMO1) cannot bind to the oocyte surface. No significant (N.S.) difference was observed between the binding indexes of the cells overexpressing all of the five factors and IZUMO1 only.

We examined the conservation of SOF1, TMEM95, and SPACA6 among species using TreeFam (http://www.treefam.org/). TreeFam is a database indicating the phylogenetic trees predicted from animal genomes. This software also provides orthology/paralogy predictions as well as the evolutionary histology of genes (36). These genes were rarely conserved in animal species, such as flies, frogs, and birds, but they were widely conserved in mammals (Fig. 6*A*). Specifically, these genes were conserved in rodents, livestock (e.g., pig and cow), and primates, including human (*SI Appendix*, Fig. S10). These results are similar to the phylogenetic trees of FIMP and IZUMO1 (Fig. 6*A* and *SI Appendix*, Fig. S10). Thus, *Sof1*, *Tmem95*, and *Spaca6* emerged as fusion-related genes in gametes during evolution.

### In Vitro Cell-Based Assays Depicted Codependency of Fusion-Related Proteins.

To assess potential interactions between IZUMO1 and other fusion-related proteins, co-IP was carried out using the lysates of HEK293T cells coexpressing IZUMO1 and each of the other fusion-related proteins. IZUMO1 was found to interact with SOF1 (*SI Appendix*, Fig. S11*A*), TMEM95, SPACA6 (*SI Appendix*, Fig. S11*B*), and FIMP (*SI Appendix*, Fig. S11*C*) in HEK293T cells. ADAM1B, a protein that localizes to the sperm surface and is not involved in sperm−oocyte fusion, was analyzed as a negative control and failed to interact with IZUMO1 as expected (*SI Appendix*, Fig. S11*D*).

Previous studies demonstrated that transient expression of recombinant mouse IZUMO1 in cultured cells, such as HEK293T and Cos-7 cells, endows them with strong binding affinity to the surface of ZP-free oocytes (17). As expected, HEK293T cells overexpressing IZUMO1-mCherry bound to the ZP-free oocytes with a binding index of 3.3 ± 1.3 cells per oocyte (Fig. 6*B*). Nevertheless, HEK293T cells expressing SOF1-1D4, TMEM95-1D4, or SPACA6-1D4 barely bind to ZP-free oocytes (Fig. 6*B*). Coexpression of IZUMO1-mCherry, SOF1-1D4, TMEM95-1D4, SPACA6-1D4, and FIMP-FLAG in HEK293T cells allowed the cells to bind to but not fuse with the oocyte membrane (Fig. 6*C*). Cultured cells overexpressing all of these proteins except for IZUMO1-mCherry failed to bind to ZP-free oocytes. Further, the binding index of HEK293T cells expressing all fusion proteins (4.0 ± 0.6 cells per oocyte) was comparable to that of the ones solely expressing IZUMO1 (3.5 ± 0.2 cells per oocyte), implying that coexpression of the other four proteins could not promote IZUMO1-derived membrane adhesion in vitro (Fig. 6*C*). Expression of the recombinant proteins in HEK293T cells was confirmed by immunoblot analysis and immunostaining (*SI Appendix*, Fig. S12).

## Discussion

Mammalian fertilization has been studied for decades, and researchers in the field have tried to discover essential factors to elucidate the molecular mechanisms underlying sperm−oocyte fusion (1). While a combination of IVF and fusion-inhibiting antibodies had nominated critical proteins, IZUMO1 was, until now, the only sperm protein proven to be essential by KO mouse studies (2, 4). Recently, by making KO mice using the CRISPR-Cas9 system, we identified sperm protein FIMP as a sperm−oocyte fusion-related protein (31). Specifically, FIMP localizes to the equatorial segment of acrosome-intact spermatozoa, and a faint signal remains after the acrosome reaction in some spermatozoa. Surprisingly, the amount and localization of IZUMO1 in *Fimp* KO spermatozoa were not influenced. Because IZUMO1 and FIMP fail to show fusogenic features, more factors need to be discovered to elucidate the sperm−oocyte fusion process. In the present study, we demonstrate that testis-enriched proteins SOF1, TMEM95, and SPACA6 are essential for sperm−oocyte fusion in mice.

SOF1 was detected as a protein singlet and a doublet in TGCs and acrosome-intact spermatozoa, respectively (Fig. 1*C*), suggesting SOF1 undergoes posttranslational modification during sperm maturation. The upper band of SOF1 remains whereas the lower band disappears after the acrosome reaction (Fig. 2*D*). Recombinant expression of SOF1 in HEK293T cells also appears as a protein doublet (*SI Appendix*, Fig. S11). These findings imply that 1) the upper form that remains in acrosome-reacted spermatozoa functions during gamete fusion or 2) the lower form is released from sperm surface during acrosomal exocytosis to activate or unmask other fusion-inducing factors. Future characterization of SOF1 is necessary to unveil its detailed functions.

Similar to IZUMO1 and SPACA6 (9, 34), TMEM95 is also identified as a type I single-pass transmembrane protein carrying a signal peptide at the N terminus and a transmembrane helix near its C terminus. It has been reported that a nonsense mutation in TMEM95, which induces truncation at the transmembrane region, leads to idiopathic subfertility in bulls (32). Bovine spermatozoa bearing the mutant TMEM95 are able to undergo the acrosome reaction but show impaired ability to fuse with the oocyte plasma membrane. Hence, the transmembrane domain may be important for the normal functioning of TMEM95 in underpinning sperm−oocyte fusion. The necessity of the transmembrane domain in fusion-related sperm factors has also been demonstrated in FIMP (31). The impaired fusion ability of *Fimp* KO spermatozoa is restored by Tg expression of the membrane isoform of FIMP.

As reported previously, TMEM95 is localized to the acrosome cap of the bull spermatozoa before acrosome reaction. Intriguingly, TMEM95 disappears in acrosome-reacted bovine spermatozoa (33). The removal of this protein during acrosome exocytosis implies that it may serve functions that are completely different from IZUMO1. TMEM95 may not act as a fusogenic protein that directly mediates the membrane fusion between spermatozoa and oocytes. Instead, it may mask the real fusogens that mediate sperm−oocyte membrane fusion before the acrosome reaction; the release of TMEM95 may facilitate the functional domain of the fusogenic protein to be exposed to the cell surface, which is required for fusion to occur. Alternatively, it is possible that sperm−oocyte membrane fusion is a bilateral fusion process, where the fusogen needs to be presented on both membranes; during acrosomal exocytosis, TMEM95 may be released and transferred to the oocyte plasma membrane, thereby ensuring fusion to occur bilaterally.

SPACA6 has been reported years ago as a sperm protein that may be required for sperm−oocyte membrane fusion (34). However, little attention has been given to this protein by researchers in the field, and the mechanism underlying this fusion-related factor remains unknown. Although the peptide sequences of SPACA6 and IZUMO1 barely share homology, profound similarities are identified between their domain compositions (*SI Appendix*, Fig. S7*B*). Thus, it is tempting to speculate that SPACA6 would exhibit IZUMO1-like function and behavior during fertilization. Utilizing a Tg mouse line expressing 1D4-tagged SPACA6, we localized SPACA6 in the spermatozoa before and after the acrosome reaction. Similar to IZUMO1 and TMEM95, SPACA6 initially localizes to the acrosome cap in acrosome-intact spermatozoa.

Distinct from IZUMO1, which is stochastically translocated to the equatorial segment or the entire sperm head after the acrosome reaction, SPACA6 spreads out only to the equatorial segment in acrosome-reacted spermatozoa. This relatively stringent translocation of SPACA6 may explain why membrane fusion usually occurs at the equatorial segment of spermatozoa (10). Although Tg expression of tagged SPACA6 facilitates the determination of the subcellular localization of this protein, the fluorescence signal of SPACA6-1D4 in the spermatozoa is very weak, which is in concert with the weak band of SPACA6-1D4 detected in sperm lysate by immunoblot analysis (Fig. 5*C*). Whether the low protein amount reflects the behavior of endogenous SPACA6 or is due to unstable Tg expression can be clarified in future studies utilizing a good antibody against mouse SPACA6.

Notwithstanding the structural similarities between IZUMO1 and SPACA6, no interaction was detected between these two proteins by co-IP tandem immunoblot analysis of testis extracts (Fig. 5*F*). However, co-IP analyses using HEK293T cells demonstrated interactions between IZUMO1 and all other known fusion-related proteins (*SI Appendix*, Fig. S11). In spermatozoa, such protein−protein interactions might be transiently established after IZUMO1 translocation upon acrosome reaction, or even after IZUMO1 binds to JUNO. Thus, it would be difficult to detect such interactions in testis or spermatozoa by co-IP. Nevertheless, expression and localization of SPACA6 and other fusion-related sperm proteins can be examined in *Izumo1* KO spermatozoa to shed light on their potential relationships during fertilization.

Cultured cells overexpressing IZUMO1 can tightly attach to the plasma membrane of mouse oocytes (16, 17, 37). Such adhesion is believed to originate from the interaction between IZUMO1 overexpressed on the plasma membrane of HEK293T cells and the receptor of IZUMO1, JUNO, on the oocyte membrane. In comparison, according to our present study, transient expression of other fusion-related sperm proteins does not bestow HEK293T cells with the ability to adhere to the oocyte surface. Thus, it is possible that FIMP, SOF1, TMEM95, and SPACA6 do not possess a counterpart on the oocyte plasma membrane, or the interactions between the ligands and receptors are too weak to recapitulate in vitro. HEK293T cells expressing all five proteins did not show improved binding or an ability to fuse with ZP-free oocytes. Thus, these five proteins are not sufficient for fusion to occur. Nevertheless, it is conceivable that membrane fusion between spermatozoa and oocytes is a highly orchestrated and dynamic process that requires strict timing and proper interplay of different molecules to enable the sperm head to internalize into the ooplasm. Future investigations are required to optimize the experimental conditions to recapitulate gamete fusion using cultured cells and ZP-free oocytes.

In conclusion, we discovered three genes, *Sof1*, *Tmem95*, and *Spaca6*, that encode sperm proteins required for fusion with oocytes. The amount and localization of IZUMO1 in these KO spermatozoa were normal. Overexpression of these proteins did not endow cultured cells with the ability to bind to or fuse with oocyte plasma membrane. Thus, it is tempting to speculate that SOF1, TMEM95, and SPACA6 may directly or indirectly regulate membrane fusion via an IZUMO1-independent pathway or act as fusion mediators downstream of the interaction between IZUMO1 and JUNO. This research emphasizes the multifactorial nature of the membrane fusion between spermatozoon and oocyte. The discovery of novel fusion-related proteins may have profound implications in unveiling the mechanism of gamete fusion at the molecular level.

## Materials and Methods

### Animals.

B6D2F1, C57BL/6J, and ICR mice were purchased from Japan SLC or CLEA Japan. Mice were acclimated to a 12-h-light/12-h-dark cycle. All animal experiments were approved by the Animal Care and Use Committee of the Research Institute for Microbial Diseases, Osaka University, Japan (#Biken-AP-H30-01).

### Sample Collection and Analyses.

For the multitissue expression analyses, brain, thymus, lung, heart, liver, spleen, kidney, testis, epididymis (caput, corpus, and cauda regions), seminal vesicle, prostate (mixture of dorsal, lateral, and ventral regions), coagulating gland (also known as anterior prostate), ovary, and uterus were collected from adult C57BL/6J mice. These samples were processed in TRIzol (Ambion). For Western blot analysis, TGC proteins were extracted with lysis buffer containing Triton-X 100 (50 mM NaCl, 10 mM Tris⋅HCl, 1% [vol/vol] Triton-X 100 [Sigma Aldrich], pH 7.5) containing 1% (vol/vol) protease inhibitor mixture (Nacalai Tesque). Proteins of cauda epididymal spermatozoa were extracted with sample buffer containing β-mercaptoethanol (Nacalai Tesque) as described previously (38).

### RT-PCR for Tissue Expression Analysis.

The total RNA was reverse-transcribed to cDNA using a SuperScript III First-Strand Synthesis System for RT-PCR (Invitrogen). PCR conditions with primer sets (*SI Appendix*, Table S1) and KOD DNA Polymerase (KOD-Fx neo; TOYOBO) were 94 °C for 3 min, denaturing at 94 °C for 30 s, annealing at 65 °C for 30 s, and elongation 72 °C for 30 s for 35 cycles in total, followed by 72 °C for 2 min.

### Generation of KO Mice.

The generation of genetically modified mice was done as described previously (26, 39). Specifically, *Sof1 del/del* mice were produced by introducing *pX459* plasmid (https://www.addgene.org/62988/) into mouse ES cells (EGR-G01) as described previously (28, 39). The mutant ES cells were injected into morula-stage embryos of the ICR strain. The germ line transmission was confirmed by mating the obtained chimeric males with B6D2F1 females. *Tmem95 del/del* and *Spaca6 del/del* mice were generated by introducing gRNA/CAS9 protein solution into fertilized eggs (B6D2 background) with an electroporator (Nepagene) (26). A search for gRNA and off-target sequences was performed using CRISPRdirect software (https://crispr.dbcls.jp/) (40) and Benchling (https://www.benchling.com/academic/). The screening of the obtained mutant mice was performed by direct sequencing following PCR. The gRNAs and primers used are listed in *SI Appendix*, Table S2. Detailed genotype information of mutant mouse lines is shown in *SI Appendix*, Figs. S2, S4, and S7.

### Generation of Tg Mice.

Sequences of mouse *Sof1* cDNA-PA tag, mouse *Tmem95* cDNA-1D4 tag, and mouse *Spaca6* cDNA-1D4 tag with a rabbit polyA signal were inserted under the control of the mouse *Clgn* promoter (*SI Appendix*, Figs. S2*G*, S6*A*, and S9*A*). The linearized DNA was injected into a pronucleus of fertilized eggs, and injected eggs were transferred to pseudopregnant females. After 19 d, offspring were obtained by natural birth or Caesarean section. Offspring carrying the transgene were screened by PCR (*SI Appendix*, Table S3).

### In Vivo Fertilization.

Pregnant mare serum gonadotropin (5 units; ASKA Pharmaceutical) or Center for Animal Resources and Development (CARD) HyperOva (0.1 mL; Kyudo) was injected into the abdominal cavity of B6D2F1 females, followed by natural mating with mutant male mice 12 h after human coagulating gland (hCG; 5 units, ASKA Pharmaceutical) injection. After 6 h to 9 h of mating, we observed the collected oocytes.

### Mating Test.

KO male mice were caged with two or three B6D2F1 females for more than 1 mo. After the mating period, the male mice were withdrawn from the cages, and the females were kept for another 20 d to allow them to deliver any possible final litters. Frozen spermatozoa from *Sof1*, *Tmem95*, and *Spaca6 del/wt* males (STOCK *Llcfc1*<*em2*Osb>, RBRC#10352, CARD#2722; STOCK *Llcfc1*<*em3*Osb>, RBRC#10353, CARD#2723; B6D2-*Spaca6*<*em1*Osb>, RBRC#10325, CARD#2695; and B6D2-*Tmem95*<*em1*Osb>, RBRC#11046, CARD#2953) will be available through RIKEN BRC (http://en.brc.riken.jp/index.shtml) and CARD R-BASE (http://cardb.cc.kumamoto-u.ac.jp/transgenic/).

### Sperm Morphology and Motility and IVF of Cauda Epididymal Spermatozoa.

Cauda epididymal spermatozoa were dispersed in phosphate-buffered saline (PBS) (for sperm morphology) or TYH drops (for sperm motility and IVF) (41). After an incubation period of 10 and 120 min in TYH, sperm motility patterns were examined using the CEROS I and II sperm analysis system (42–44). IVF was performed as described previously (45).

### Fusion Assay.

The fusion assay was performed as described previously (9). Specifically, cauda epididymal spermatozoa were incubated in TYH drops for 2 h to 3 h before the fusion assay. Unfertilized oocytes were collected from hormone-treated females 14 h after hCG injection. To remove the ZP, oocytes were treated with 1 mg/mL collagenase (C1639; Sigma Aldrich) for 10 min. After washing in TYH, oocytes were stained with Hoechst 33342 (1:10,000) for 10 min and washed repeatedly in clean TYH drops. For insemination, 1 × 10^5^ spermatozoa per mL were used. After 30 min of incubation, oocytes were fixed with 0.25% glutaraldehyde (Wako) or 0.2% paraformaldehyde (PFA).

### Antibodies.

A rat monoclonal antibody against mouse SOF1 (amino acid #22-#89, ENSMUST00000031900) was generated as described in *SI Appendix*. Monoclonal antibodies for SLC2A3 (KS64-10), IZUMO1 (KS64-125), and ADAM1B (KS107-158) were generated in our laboratory (46, 47). The anti-1D4 antibody was generated using a hybridoma cell line as a gift from Robert Molday, Ophthalmology and Visual Sciences, Centre for Macular Research, University of British Columbia, Vancouver, British Columbia, Canada (48). A polyclonal antibody for BASIGIN was purchased from Santa Cruz Biotechnology (sc-9757). A monoclonal antibody against β-Actin (ACTB) was purchased from Abcam (ab6276). A monoclonal and a polyclonal antibody against FLAG were purchased from Sigma Aldrich (F1804) and MBL (PM020), respectively. Horseradish peroxidase (HRP)-conjugated goat anti-rat immunoglobulins (IgGs) (112-035-167), HRP-conjugated goat anti-mouse IgGs (115-036-062), and HRP-conjugated bovine anti-goat IgGs antibodies (805-035-180) were purchased from Jackson ImmunoResearch Laboratories. Fluorophore-conjugated secondary antibodies goat-anti rat IgG Alexa Fluor 488 (A11006), goat-anti rat IgG Alexa Fluor 546 (A11081), goat-anti mouse IgG Alexa Fluor 488 (A11017), and goat-anti mouse IgG Alexa Fluor 546 (A11018) were purchased from Thermo Fisher Scientific.

### Western Blot Analysis.

Before sodium dodecyl sulfate polyacrylamide gel electrophoresis (SDS/PAGE), all samples were mixed with sample buffer containing β-mercaptoethanol (38) and boiled at 95 °C for 5 min. Poly(vinylidene difluoride) membrane was treated with Tris-buffered saline (TBS)-0.1% Tween 20 (Nacalai Tesque) containing 10% skim milk for 1 h, followed by the primary antibody (nondilution [SOF1], 1:1,000 [SLC2A3, IZUMO1, ACTB, FLAG, and BASIGIN], 1:3,000 [1D4]) for 3 h at room temperature or overnight at 4 °C. After washing with TBS-0.1% Tween 20, the membrane was further probed with the secondary antibody (1:1,000 [SOF1, SLC2A3, IZUMO1, ACTB, FLAG and 1D4], 1:5,000 [BASIGIN]) for 1 h. The protein bands were visualized by Amersham ECL Prime Western blot detection reagent (GE Healthcare).

### Immunocytochemistry.

After 10 min or 2 h of incubation in TYH drops, spermatozoa were pelleted by centrifugation, washed in PBS, and smeared and dried on microscope slides. The samples were fixed with 1% PFA, followed by permeabilizing with 1% Triton X-100 (Sigma Aldrich). The spermatozoa were then blocked with 10% goat serum (Gibco) for 1 h. The spermatozoa were subjected to immunostaining with anti-IZUMO1 or anti-1D4 antibody (1:200). Goat-anti rat or mouse IgG Alexa Fluor 488 or 546 (1:300) was used as the secondary antibody. HEK293T cells were seeded on coverslips coated with poly-l-lysine (Sigma Aldrich) and transfected with different expression vectors encoding proteins of interest. The cells were fixed in 4% PFA, permeabilized with 0.1% Triton X-100, and blocked with 10% goat serum for 1 h. The cells were then probed with anti-1D4 or anti-FLAG antibody (1:200), followed by secondary antibody goat-anti mouse IgG Alexa Fluor 546 (1:200).

### Ca^2+^ Ionophore A23187 Treatment.

A23187 treatment was performed as described previously to induce the acrosome reaction in cauda epididymal spermatozoa (49). Specifically, spermatozoa from ICR male mice were preincubated in a TYH drop for 1 h. The spermatozoa were subsequently incubated in a TYH drop containing 10 μM A23187 (Merck) or 0.5% (vol/vol) dimethyl sulfoxide (DMSO) (Sigma Aldrich) for 1 h. After washing with PBS, stimulation of the acrosome reaction in the spermatozoa was confirmed by the translocation of IZUMO1, and the spermatozoa were subjected to Western blot analysis.

### Co-IP.

Testis or HEK293T cell lysates, extracted using lysis buffer (50 mM Tris [pH 7.5], 150 mM NaCl, 1% Triton X-100, 10% Glycerol, 1 mM dithiothreitol, and 1% protease inhibitor mixture in distilled water), were incubated with antibody-conjugated Pierce Protein A/G Magnetic Beads (Thermo Fisher Scientific) for 1 h at 4 °C. After washing three times with wash solution (50 mM Tris [pH 7.5], 150 mM NaCl, 0.1% Triton X-100, and 10% Glycerol in distilled water), protein complexes were eluted with SDS sample buffer supplemented with 5% β-mercaptoethanol and denatured at 70 °C for 10 min. Proteins were then separated by SDS/PAGE and analyzed by Western blot analysis.

### HEK293T−Oocyte Binding Assay.

*Izumo1*, *Sof1*, *Tmem95*, *Spaca6,* and *Fimp* ORFs were cloned from mouse testis cDNA, a Kozak sequence was added upstream of the ATG start codon, and a sequence containing an epitope tag was added in-frame prior to the stop codon, using RT-PCR. The PCR amplicons were inserted into the pCAG1.1 vector bearing a CAG promoter and a rabbit beta-globin polyadenylation signal. HEK293T cells were transfected with expression vectors by calcium phosphate−DNA coprecipitation method (39). After 2 d of incubation, transfected HEK293T cells were resuspended in PBS containing 10 mM (ethylenedinitrilo)tetraacetic acid and stained with Hoechst 33342. ZP-free oocytes were collected as described above. The ZP-free oocytes were incubated with HEK293T cells overexpressing different proteins of interest in TYH for 2 h and observed under a Nikon Eclipse Ti confocal laser scanning microscope. Oocytes were subsequently incubated in potassium simplex optimization medium for a longer period to find out whether the cultured cells would fuse with the oocyte plasma membrane.

### Statistical Analyses.

All values are shown as the mean ± SD of at least three independent experiments. Statistical analyses were performed using the Student’s *t* test, after examining normal distribution and variance.

### Data Availability Statement.

All data are included in the paper or *SI Appendix*.

## Supplementary Material

Supplementary File

Supplementary File

Supplementary File

Supplementary File

Supplementary File

Supplementary File
