# Supplementary material for: Sperm proteins SOF1, TMEM95, and SPACA6 are required for sperm−oocyte fusion in mice
Source: Proc Natl Acad Sci U S A. 2020 May 11;117(21):11493–502. doi: 10.1073/pnas.1922650117 (PMC7261011; doi:10.1073/pnas.1922650117)
Supplement: Supplementary File [file pnas.1922650117.sapp.pdf]

PNAS
www.pnas.org

Supplementary Information for

Sperm proteins SOF1, TMEM95, and SPACA6 are required for sperm-oocyte fusion in mice

Taichi Noda[1,#], Yonggang Lu[1,#], Yoshitaka Fujihara[1,*], Seiya Oura[1,2], Takayuki Koyano[3], Sumire Kobayashi[1,2], Martin M. Matzuk[4,**], and Masahito Ikawa[1,5,**]

[1]Research Institute for Microbial Diseases, Osaka University, 3-1 Yamadaoka, Suita, Osaka 565-0871, Japan. [2]Graduate School of Pharmaceutical Sciences, Osaka University, 1-6 Yamadaoka, Suita, Osaka 565-0871, Japan. [3]Division of Molecular Genetics, Shigei Medical Research Institute, 2117 Yamada, Minami-ku, Okayama, 701-0202, Japan. [4]Center for Drug Discovery and Department of Pathology & Immunology, Baylor College of Medicine, One Baylor Plaza, Houston, TX 77030, USA. [5]The Institute of Medical Science, The University of Tokyo, 4-6-1 Shirokanedai, Minato-ku, Tokyo 108-8639, Japan

[#]T.N., and Y.L. contributed equally to this work
*Present address: Department of Bioscience and Genetics, National Cerebral and Cardiovascular Center, Suita, Osaka 564-8565, Japan
**Correspondences and requests for materials should be addressed to M.M.M. (email: mmatzuk@bcm.edu) or M.I. (email: ikawa@biken.osaka-u.ac.jp)

This PDF file includes:

Supplementary text
Figures S1 to S12
Tables S1 to S3
Legends for Movies S1 to S5
SI References

www.pnas.org/cgi/doi/10.1073/pnas.1922650117

Supplementary Information Text

Single-cell RNA-sequencing data

mRNA expression of *Fimp*, *Izumo1*, *Sof1*, *Spaca6* and *Tmem95* in spermatogenic cells was examined *in silico* by 10x Genomics software with single-cell RNA-sequencing dataset published previously (1).

Sequence alignment and phylogenetic trees

Multiple sequence alignments and gene trees were made by GENETYX with the amino acid sequences of the proteins of interest [for FIMP: ENSCAFT00000036402 (dog), ENSSSCT00000062827 (pig), ENSBTAT00000083974 (cow), ENSPTRT00000014730 (chimpanzee), ENST00000300575 (human), ENSMUST00000061695 (mouse), ENSRNOT00000047477 (rat), ENSMAUT00000013394 (golden hamster)] [for IZUMO1: ENSDART00000184278 (zebrafish), XP_022279896 (dog), ENSSSCT00000042201 (pig), ENSBTAT00000015434 (cow), ENSPTRT00000020910 (chimpanzee), ENST00000332955 (human), ENSOCUT00000030458 (rabbit), ENSMUST00000033100 (mouse), ENSRNOT00000033278 (rat), ENSMAUT00000015944 (golden hamster)] [for SOF1: ENSCAFT00000043861 (dog), ENSSSCT00000065516 (pig), XP_005228669 (cow), ENSPTRT00000073443 (chimpanzee), ENST00000409607 (human), ENSOCUT00000007564 (rabbit), ENSMUST00000031900 (mouse), ENSRNOT00000031984 (rat), ENSMAUT00000021002 (golden hamster)] [for SPACA6: XP_021322589 (zebrafish), ENSCAFT00000043610 (dog), ENSSSCT00000003597 (pig), ENSBTAT00000065736 (cow), ENSPTRT00000092083 (chimpanzee), ENST00000637797 (human), XP_017193772 (rabbit), ENSMUST00000172097 (mouse), ENSRNOT00000014598 (rat), ENSMAUT00000004923 (golden hamster)] [for TMEM95: ENSCAFT00000078659 (dog), ENSSSCT00000019529 (pig), ENSBTAT00000080171 (cow), ENSPTRT00000015998 (chimpanzee), ENST00000389982 (human), ENSOCUT00000028924 (rabbit), ENSMUST00000178597 (mouse), ENSRNOT00000065524 (rat)]. The neighbor-joining method was used for each gene tree.

Histology

Testis and epididymis were fixed in Bouin's fluid (Polysciences) at 4°C overnight. Fixed samples were dehydrated by increasing ethanol concentrations and then were embedded with paraffin. Paraffin sections (5 μm) were stained with 1% periodic acid solution (Wako) for 10 minutes, followed by treatment with Schiff's reagent (Wako) for 20 minutes, and then Mayer's hematoxylin solution (Wako) for 5 minutes. After dehydration with ethanol, these slides were mounted with Entellan® new (Merck Millipore) and observed with phase contrast microscopy.

Production of a monoclonal antibody against mouse SOF1

The DNA encoding mouse SOF1 (residue 22-89 aa, ENSMUST00000031900) were inserted into pGEX6p-1 (GE healthcare), and the expression vector was transformed into *E. coli* strain BL21 (de3) pLysS (C606003, Thermo Fisher Scientific). Expression of GST-fused SOF1 was induced by adding IPTG (Nacalai Tesque) to final concentration of 1.0 mM. After centrifugation, the cell pellet was suspended in Lysis buffer [150 mM

NaCl, 50 mM Tris-HCl, 2% (v/v) Triton X-100, 1 mM DTT, 100 µg/mL Lysozyme, protease inhibitor cocktail tablets (Merck) , pH 8.0] and lysed by ultrasonic disruptor (UD-201, TOMY). After centrifugation, the supernatant was incubated with Glutathione Sepharose 4B (GE Healthcare) for 1 hour with gentle agitation. The lysate was loaded on a column and washed with 20 mL of wash buffer A (50 mM KCl, 20 mM $MgCl_2$, 50 mM Tris-HCl, 5 mM ATP, pH 7.4), followed by 20 mL of wash buffer B (wash buffer A without ATP). GST-fused SOF1 was eluted from the column with elution buffer (150 mM NaCl, 50 mM Tris-HCl, 10 mM glutathione, pH 9.5). PreScission protease (GE Healthcare) was added into GST-fused SOF1-containing fractions for GST cleavage, and the samples were dialyzed against reaction buffer (150 mM NaCl, 50 mM Tris-HCl, 1 mM EDTA, 1 mM DTT, pH 7.0) for 16 hours. The enzymatically-cleaved GST and PreScission protease were removed by Glutathione Sepharose 4B affinity subtraction purification. Recombinant SOF1 solution was concentrated using Vivaspin (GE Healthcare) followed by buffer exchange into PBS using PD10 column (GE Healthcare). Recombinant SOF1 was used to produce the monoclonal antibody as previously described (2). Specifically, purified SOF1 protein with a complete adjuvant was injected into female rats. After 17 days of injection, lymphocytes were collected from iliac lymph nodes and hybridomas generated (3). Culture supernatants of hybridomas were used as antibodies. The candidates were screened by ELISA against SOF1.

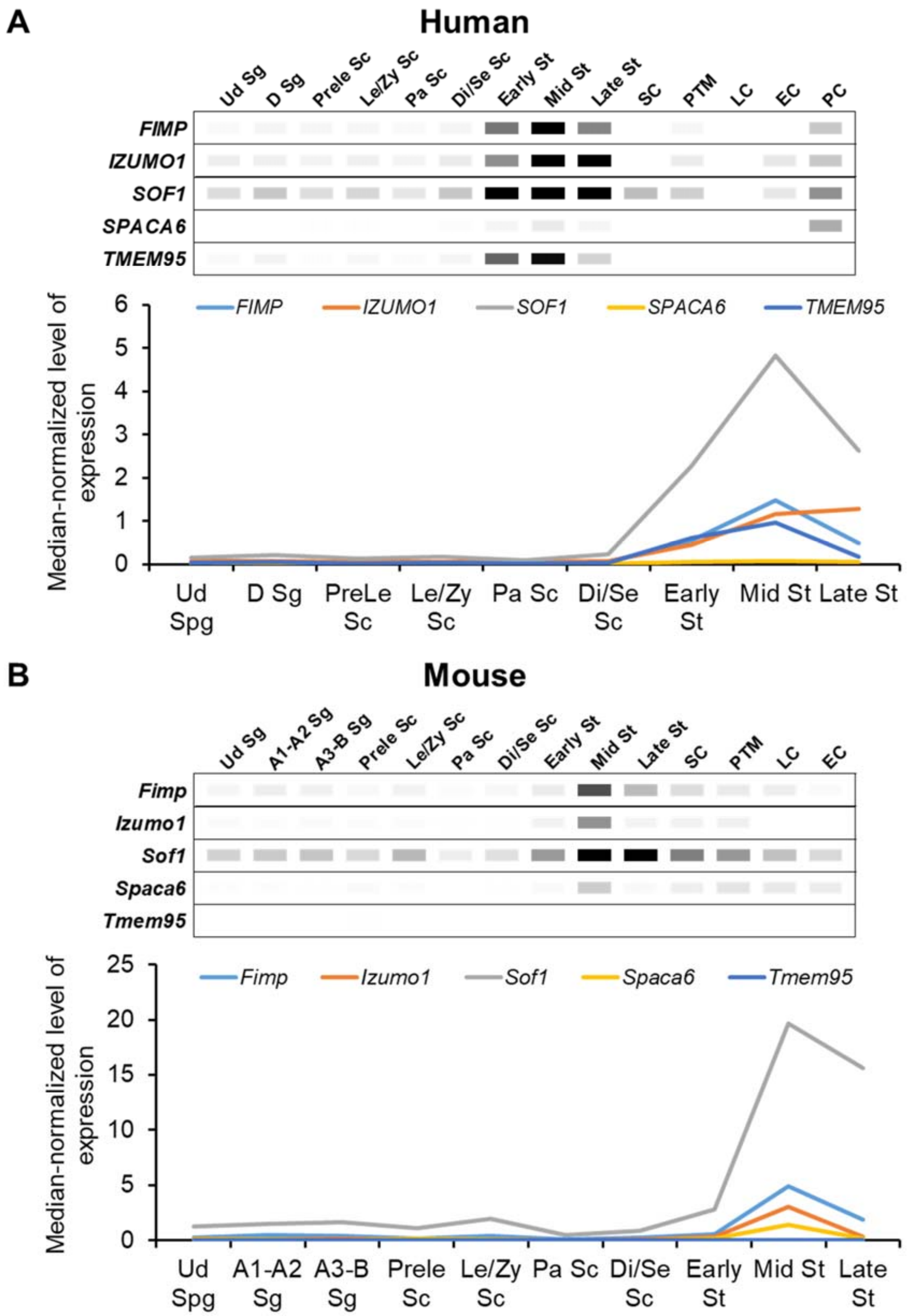
A
Human
Ud Sg
D Sg
Prele Sc
Le/Zy Sc
Pa Sc
Di/Se Sc
Early St
Mid St
Late St
SC
PTM
LC
EC
PC
FIMP
IZUMO1
SOF1
SPACA6
TMEM95
Median-normalized level of expression
Ud Spg
D Sg
PreLe Sc
Le/Zy Sc
Pa Sc
Di/Se Sc
Early St
Mid St
Late St
B
Mouse
Ud Sg
A1-A2 Sg
A3-B Sg
Prele Sc
Le/Zy Sc
Pa Sc
Di/Se Sc
Early St
Mid St
Late St
SC
PTM
LC
EC
Fimp
Izumo1
Sof1
Spaca6
Tmem95
Median-normalized level of expression
Ud Spg
A1-A2 Sg
A3-B Sg
Prele Sc
Le/Zy Sc
Pa Sc
Di/Se Sc
Early St
Mid St
Late St

Figure S1. Single-cell RNA-sequencing data depicting the expression of *Fimp*, *Izumo1*, *Sof1*, *Spaca6* and *Tmem95* during human and mouse spermatogenesis.

A) **Median-normalized level of mRNA expression of *FIMP*, *IZUMO1*, *SOF1*, *SPACA6* and *TMEM95* during spermatogenesis in human.** Ud Sg, undifferentiated spermatogonia; D Sg, differentiated spermatogonia; Prele Sc, preleptotene spermatocytes; Le/Zy Sc, leptotene/zygotene spermatocytes; Pa Sc, pachytene spermatocytes; Di/Se Sc, diplotene/secondary spermatocytes; Early St, early round spermatids; Mid St, mid round spermatids; Late St, late round spermatids; SC, Sertoli cells; PTM, peritubular myoid cells; LC, Leydig cells; EC, endothelial cells; PC, perivascular cells. Upper panel: The level of mRNA expression in each cell type is indicated by the intensity of each band. White: median-normalized reads = 0; Black: median-normalized reads ≥ 7; Gray: 0 < median-normalized reads < 7. Lower panel: A linear graph shows the mRNA expression level of each gene at various spermatogenic cell stages.

B) **Median-normalized level of mRNA expression of *Fimp*, *Izumo1*, *Sof1*, *Spaca6* and *Tmem95* during spermatogenesis in mice.** Ud Sg, undifferentiated spermatogonia; A1-A2 Sg, A1-A2 differentiating spermatogonia; A3-B Sg, A3-A4-In-B differentiating spermatogonia; Prele Sc, preleptotene spermatocytes; Le/Zy Sc, leptotene/zygotene spermatocytes; Pa Sc, pachytene spermatocytes; Di/Se Sc, diplotene/secondary spermatocytes; Early St, early round spermatids; Mid St, mid round spermatids; Late St, late round spermatids; SC, Sertoli cells; PTM, peritubular myoid cells; LC, Leydig cells; EC, endothelial cells. Upper panel: The level of mRNA expression in each cell type is indicated by the intensity of each band. White: median-normalized reads = 0; Black: median-normalized reads ≥ 1; Gray: 0 < median-normalized reads < 1. Lower panel: A linear graph shows the mRNA expression level of each gene at various spermatogenic cell stages.

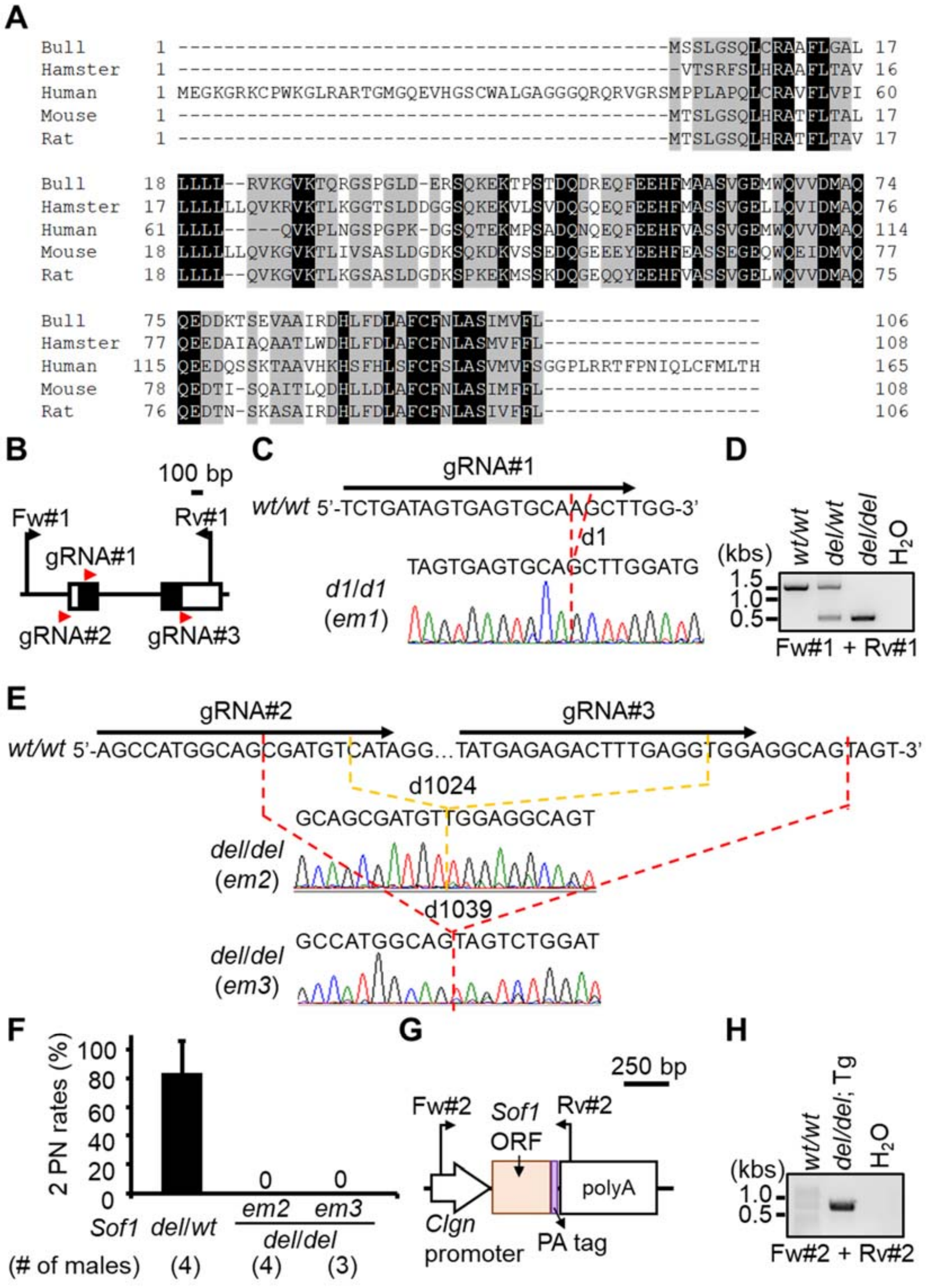
A
Bull
Hamster
Human
Mouse
Rat
B
100 bp
Fw#1
Rv#1
gRNA#1
gRNA#2
gRNA#3
C
gRNA#1
wt/wt 5'-TCTGATAGTGAGTGCAAGCTTGG-3'
d1
TAGTGAGTGCAGCTTGGATG
d1/d1
(em1)
D
wt/wt
del/wt
del/del
H2O
(kbs)
1.5
1.0
0.5
Fw#1 + Rv#1
E
gRNA#2
gRNA#3
wt/wt 5'-AGCCATGGCAGCGATGTCATAGG…TATGAGAGACTTTGAGGTGGAGGCAGTAGT-3'
d1024
GCAGCGATGTTGGAGGCAGT
del/del
(em2)
d1039
GCCATGGCAGTAGTCTGGAT
del/del
(em3)
F
2 PN rates (%)
100
80
60
40
20
0
Sof1 del/wt
em2
em3
del/del
(# of males) (4) (4) (3)
G
250 bp
Fw#2
Sof1
ORF
Rv#2
polyA
Clgn
promoter
PA tag
H
wt/wt
del/del; Tg
H2O
(kbs)
1.0
0.5
Fw#2 + Rv#2

Figure S2. Generation of *Sof1* gene deletion mice and *Sof1*-PA tag transgenic mice.

A) **Comparison of SOF1 amino acid sequence between species**. SOF1 amino acid sequences of bull (accession #: XP_005228669), hamster (ENSMAUT00000021002), human (ENST00000409607), mouse (ENSMUST00000031900), rat (ENSRNOT00000031984) were used for this analysis. Residues conserved among all displayed species are labeled in black. The ones conserved in more than half but not all of the species are highlighted in gray. SOF1 of these species conserve two sequence motif "LLLL and CFNLAS", called as DUF4717 super family using CD-search.

B) **gRNA design.** Three guide RNAs (gRNA#1-#3, red-colored arrows) were designed to target exons 1 and 2 (**SI Appendix, Table S2**). Forward (Fw) #1 and reverse (Rv) #1 primers were used for genotyping (see panel D and **SI Appendix, Table S2**). Black- and white-colored boxes show the open reading frame (ORF) and untranslated regions (UTR) of mouse *Sof1*.

C) **Direct sequencing of *Sof1* mutant mice generated with gRNA#1.** Although mice lacking 1 bp [enzyme mutation 1 (*em1*)] were obtained, *Sof1* whole deletion mice (see panel D and E) were used for phenotypic analyses.

D) **Genotyping of *Sof1* mutant mice generated with gRNA#2 and #3.** Genotyping was conducted by PCR with Fw #1 and Rv #1 primers (also see **SI Appendix, Table S2**). *wt*: wild-type, *del*: deletion.

E) **Direct sequencing of *Sof1* mutant mice generated with gRNA#2 and #3.** Mice lacking *Sof1* ORF [1024 bp (*em2*) or 1039 bp (*em3*)] were obtained.

F) **Fertilization rates of oocytes retrieved from females mated with *Sof1* mutant males.** Each male was mated with hormone treated females. After 7 hours of mating, the oocytes were collected and observed. All oocytes from females mated with *Sof1 del/del* males remained unfertilized {2 PN rates: 83.8 ± 22.0% (*del/wt*, 72 oocytes), 0% [*del/del* (*em2*), 109 oocytes, **SI Appendix, Movie S1**], and 0% [*del/del* (*em3*), 80 oocytes, **SI Appendix, Movie S2**]}. As there was no difference in the phenotype between *del/del* (*em2*) and *del/del* (*em3*), we used *del/del* (*em2*) mice for following analyses unless otherwise specified.

G) **Construct of *Sof1* transgene.** The transgene (Tg) of mouse *Sof1* and PA tag was expressed as a fused protein under the control of the testis-specific Calmegin (*Clgn*) promoter. Fw#2 and Rv#2 were used for genotyping (see panel H and **SI Appendix, Table S3**). poly A: a rabbit beta-globin polyadenylation signal.

H) **Genotyping of *Sof1*-PA Tg mice.** The genotyping was conducted by PCR with Fw#2 and Rv#2 primers (also see **SI Appendix, Table S3**).

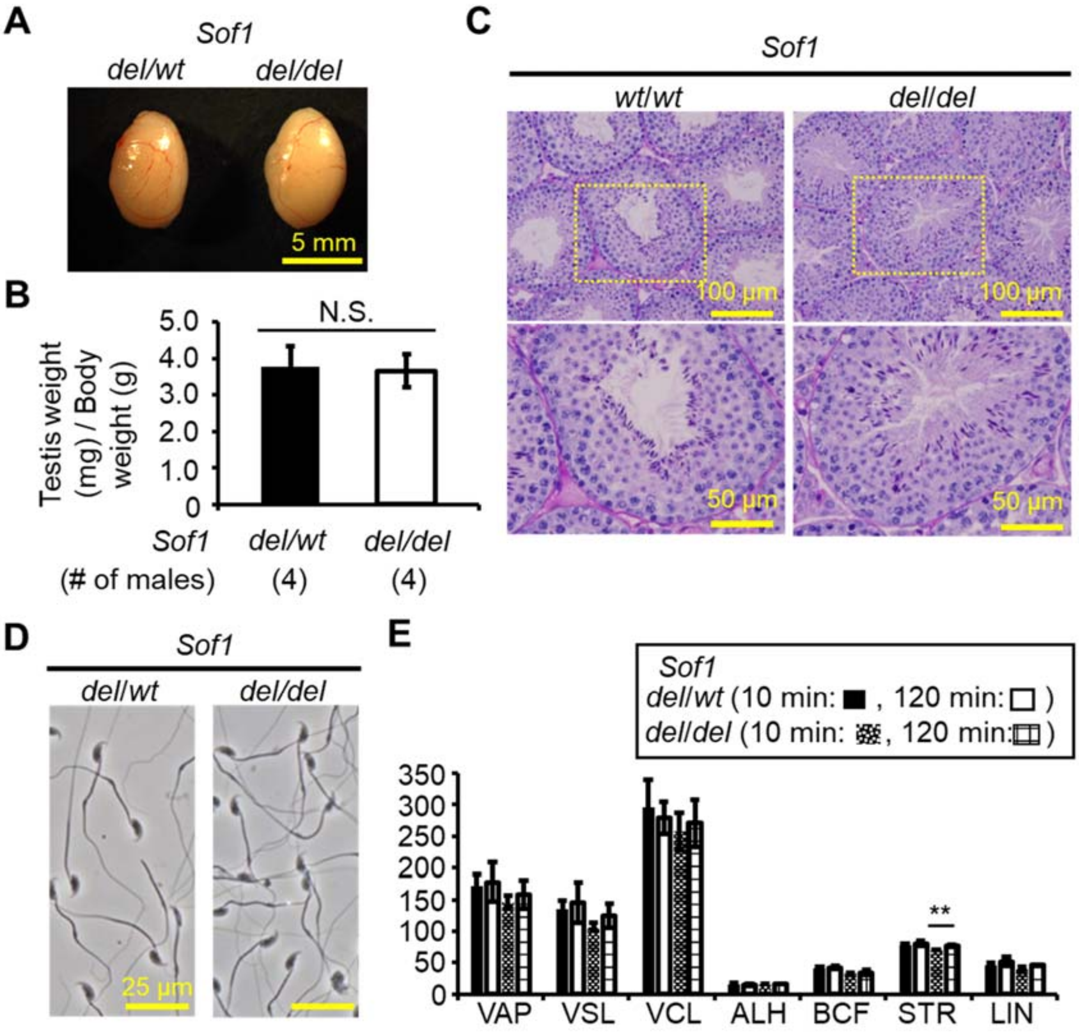

Figure S3. Analyses of *Sof1 del/del* testes and KO spermatozoa.

A) **Testis morphology**.

B) **Testis weight (mg) / body weight (g).** There was no difference between *Sof1 del/wt* and *del/del* males [testis weight / body weight: 3.76 ± 0.57 (*del/wt*, n = 4), 3.66 ± 0.45 (*del/del*, n = 4)]. N.S.: not significant.

C) **Histological analysis of testes.** Paraffin sections (5 µm) were stained by Periodic acid-Schiff (PAS)-Hematoxylin. Spermatogenesis of *Sof1 del/del* males was comparable to *Sof1 del/wt* males.

D) **Sperm morphology.** The sperm morphology of *Sof1 del/del* males was comparable to *Sof1 del/wt* males.

E) **Sperm motility parameters.** Each sperm parameter was measured using CEROS I. The overt defects of the sperm motility in *Sof1 del/del* males were not observed. VAP: average path velocity, VSL: straight line velocity, VCL: curvilinear velocity, ALH: amplitude of lateral head displacement, BCF: beat cross frequency, STR: straightness, and LIN: linearity. **: $p < 0.01$.

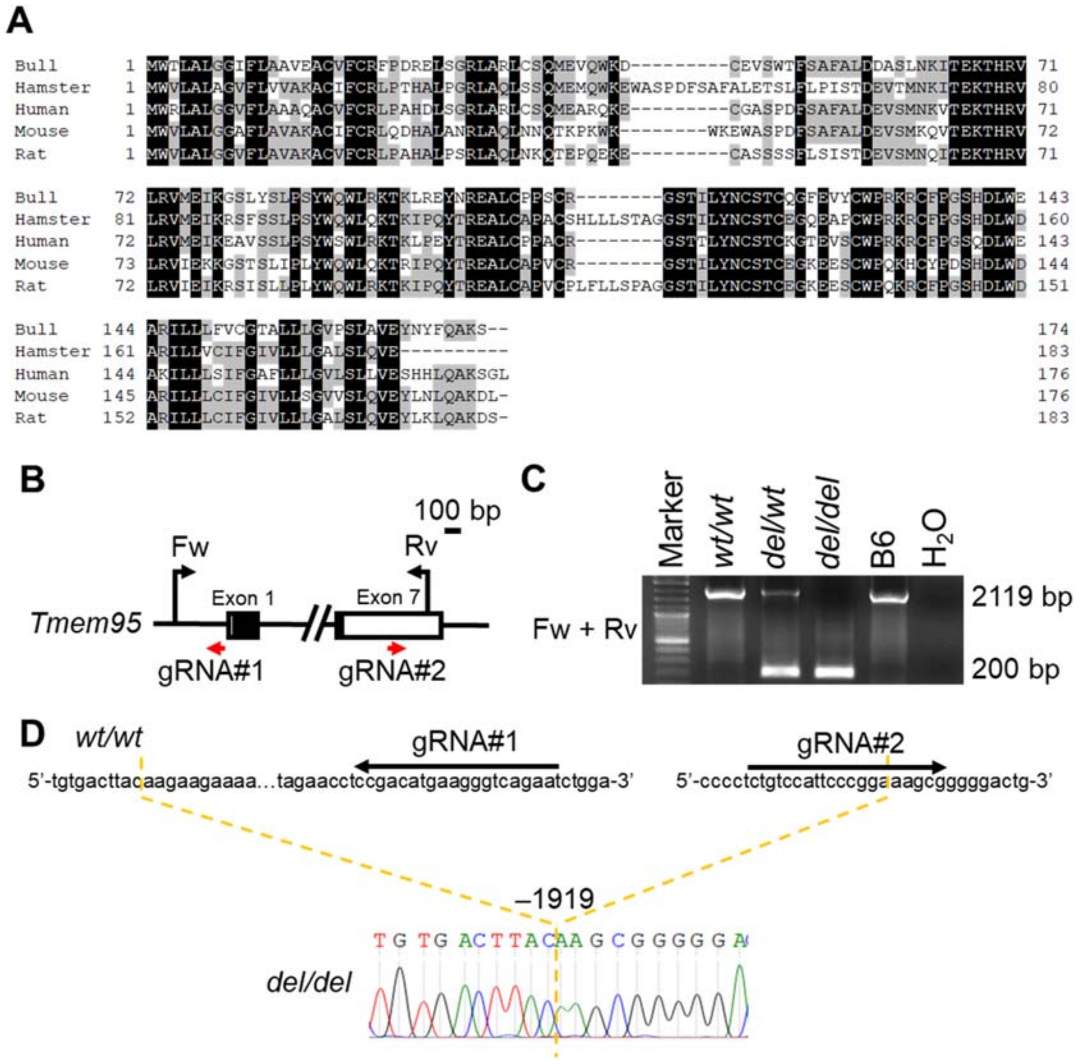

Figure S4. Generation of *Tmem95* gene deletion mice.

A) **Multiple alignment of TMEM95 peptide sequences from various species.** The protein sequences of bull, hamster, human, mouse and rat TMEM95 are aligned using the Parallel Editor of GENETYX software. Residues conserved among all displayed species are labeled in black; the ones conserved in more than half but not all of the species are highlighted in gray.

B) **Schematic presentation of *Tmem95* KO in mice using CRISPR/Cas9.** gRNA #1 and #2 were designed to target the intron upstream of the first coding exon and 3' untranslated region of the last exon, respectively (**SI Appendix, Table S2**). The Fw and Rv primers were used to detect both the *wt* and mutant alleles by PCR (**SI Appendix, Table S2**).

C) **Identification of *wt* and mutant alleles by genomic PCR.** Utilizing the Fw and Rv primers, *wt* and mutant alleles can be amplified as a 2119 bp and a 200 bp PCR product, respectively.

D) **Sanger sequencing of the mutant allele.** Sanger sequencing revealed that 1919 bp genomic region was deleted in the targeted *Tmem95* locus in the mutant mice.

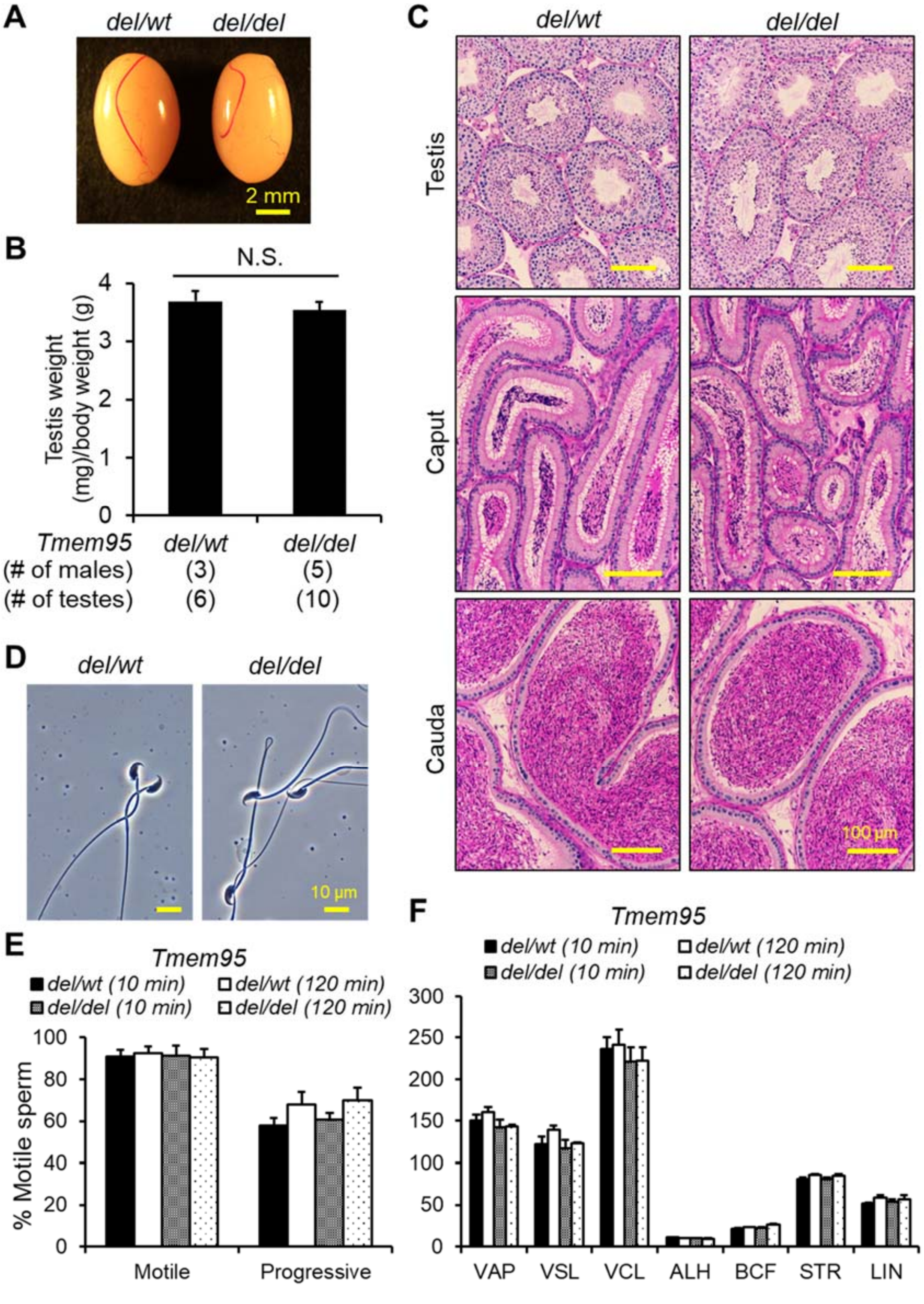

A
del/wt
del/del
2 mm
B
N.S.
Testis weight (mg)/body weight (g)
Tmem95
del/wt
del/del
(# of males)
(3)
(5)
(# of testes)
(6)
(10)
C
del/wt
del/del
Testis
Caput
Cauda
100 µm
D
del/wt
del/del
10 µm
E
Tmem95
del/wt (10 min)
del/wt (120 min)
del/del (10 min)
del/del (120 min)
% Motile sperm
Motile
Progressive
F
Tmem95
del/wt (10 min)
del/wt (120 min)
del/del (10 min)
del/del (120 min)
VAP
VSL
VCL
ALH
BCF
STR
LIN

Figure S5. Analyses of *Tmem95 del/del* testes and KO spermatozoa.

A) **Comparison of testis size and appearance in *Tmem95 del/wt* and *del/del* males.**
B) **Comparison of testis to body weight ratios in *Tmem95 del/wt* and *del/del* males.** N.S.: not significant.
C) **Histological analyses of testes and caput and cauda epididymides in *Tmem95 del/wt* and *del/del* males.** Testes and epididymides were fixed in Bouin's fluid and embedded in paraffin wax. Paraffin sections were stained by Periodic Acid Schiff and Hematoxylin.
D) **Comparison of sperm morphology in *Tmem95 del/wt* and *del/del* males.**
E) **Analyses of sperm motility and progressive movement in *Tmem95 del/wt* and *del/del* males.** Sperm motility was analyzed at 10 and 120 minutes of incubation in TYH medium using Ceros II.
F) **Analyses of sperm kinetic parameters in *Tmem95 del/wt* and *del/del* males.** Each parameter was measured at 10 and 120 minutes of incubation in TYH medium using Ceros II.

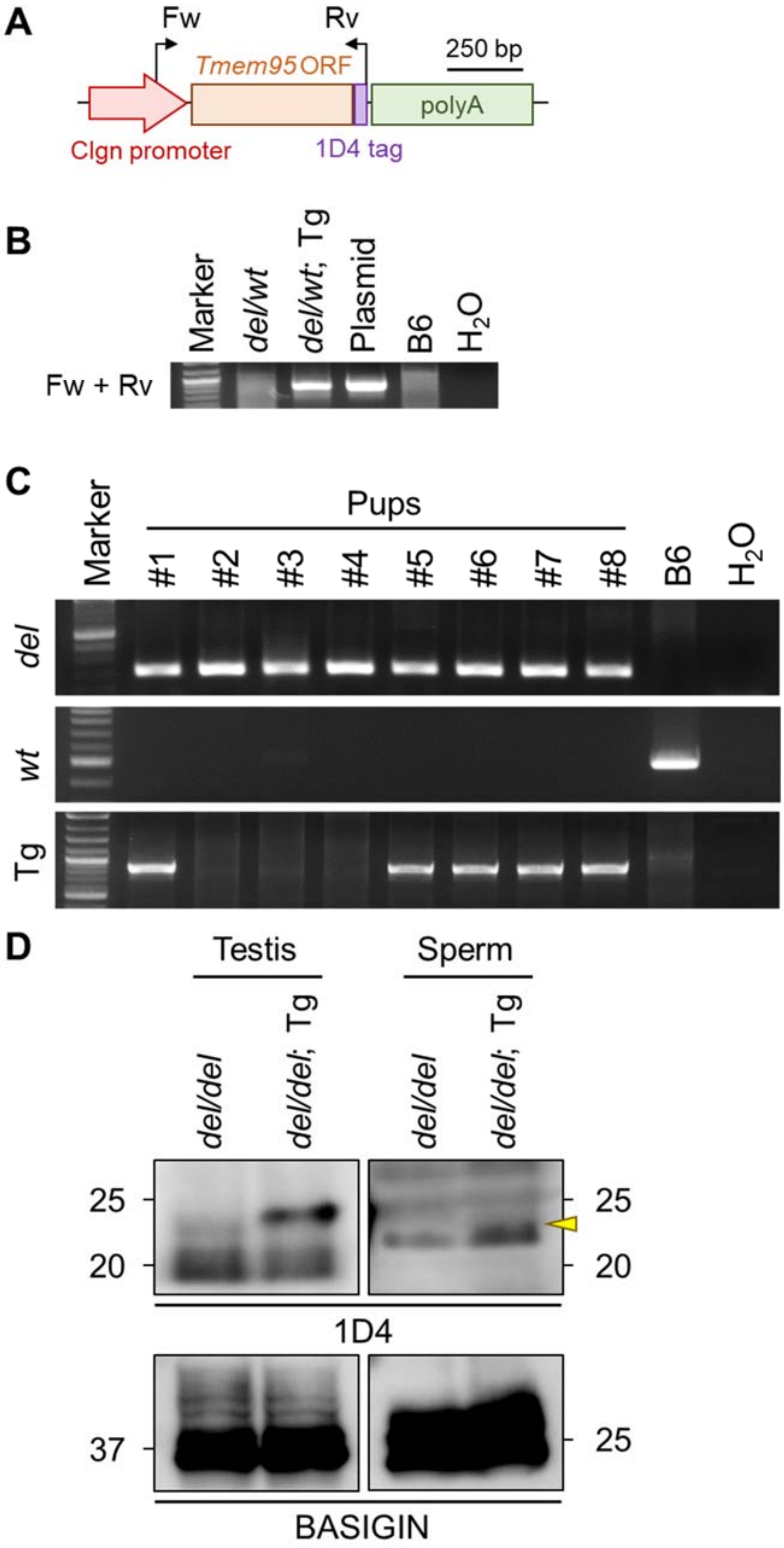
A
Fw
Rv
250 bp
Tmem95 ORF
polyA
Clgn promoter
1D4 tag
B
Marker
del/wt
del/wt; Tg
Plasmid
B6
H2O
Fw + Rv
C
Marker
Pups
#1
#2
#3
#4
#5
#6
#7
#8
B6
H2O
del
wt
Tg
D
Testis
Sperm
del/del
del/del; Tg
del/del
del/del; Tg
25
20
25
20
1D4
37
25
BASIGIN

Figure S6. Generation of *Tmem95*-1D4 Tg mice.

A) **Schematic presentation of generating *Tmem95*-1D4 Tg mice by microinjection of linearized plasmid into the pronuclei of zygotes.** The expression vector contained a *Clgn* promoter upstream of the *Tmem95* ORF and polyA downstream of an 1D4 sequence. The Fw and Rv primers were designed to identify mice bearing the transgene (see **SI Appendix, Table S3**).
B) **Identification of mice carrying the *Tmem95*-1D4 Tg by PCR.** The Fw and Rv primers were used to analyze the presence of the transgene *Tmem95*-1D4 (838 bp). The original expression vector p*Clgn*-*Tmem95*-1D4 was utilized as a positive control to indicate the expected size of PCR amplicon.
C) **Genotypic validation of offspring sired by a *Tmem95 del/del* Tg male.** A *Tmem95 del/del* female was paired with the Tg male and delivered eight pups in one litter. All of the offspring carried a *del* allele but lacked a *wt* allele, suggesting they were all homozygotes. Five of the pups were positive for the transgene, indicating successful transmission of the transgene to the next generation.
D) **Expression of TMEM95-1D4 in testes and cauda epididymal spermatozoa of Tg male mice.** Testis and sperm lysates from a *del/del* transgenic male (*del/del*; Tg) were used to test transgenic expression. Testis and sperm lysates from a *del/del* male were used as a negative control. The expression of BASIGIN was analyzed in parallel as a loading control. Low amount of TMEM95-1D4 was detected in Tg spermatozoa (yellow arrowhead).

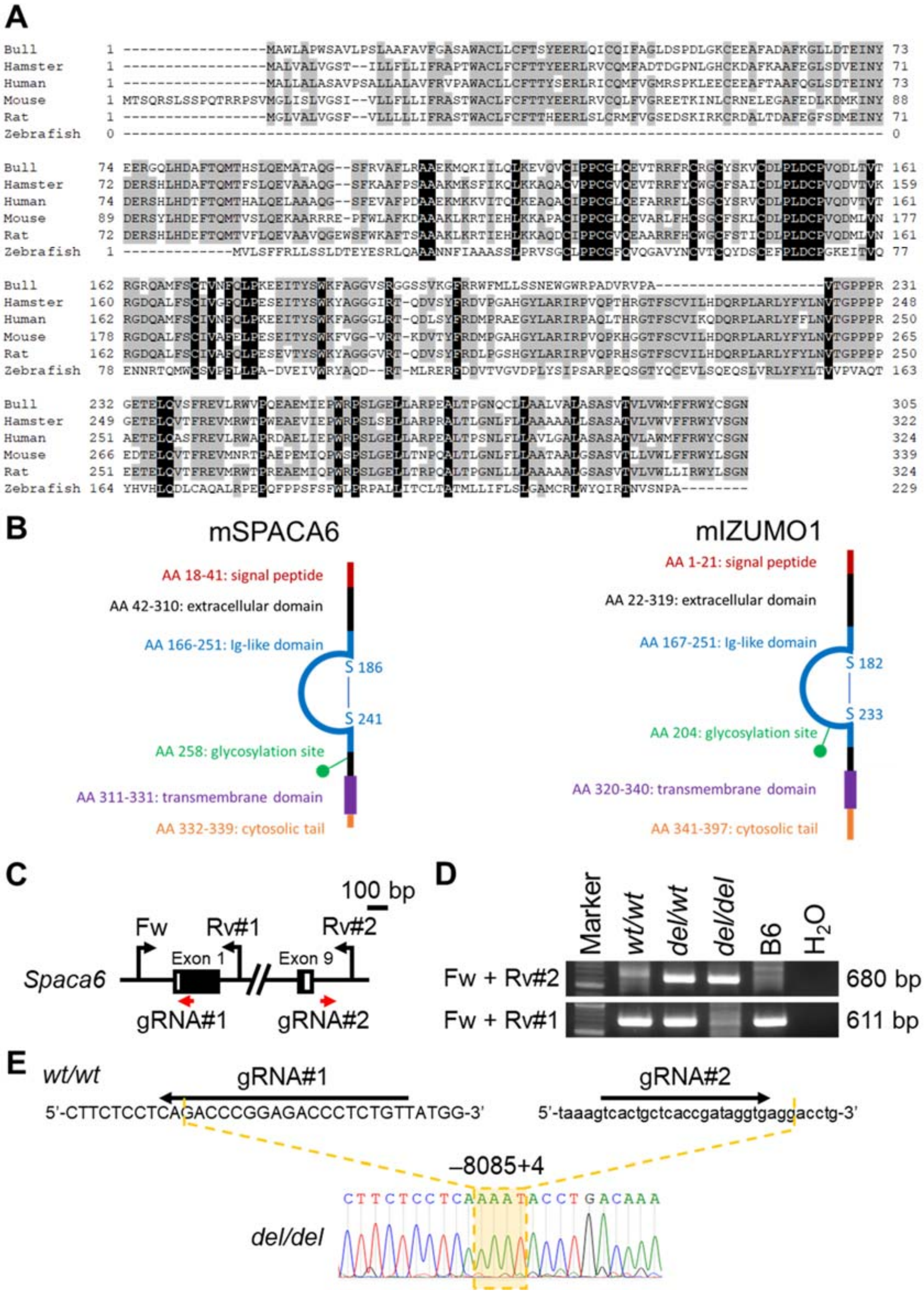
A
Bull
Hamster
Human
Mouse
Rat
Zebrafish
B
mSPACA6
AA 18-41: signal peptide
AA 42-310: extracellular domain
AA 166-251: Ig-like domain
S 186
S 241
AA 258: glycosylation site
AA 311-331: transmembrane domain
AA 332-339: cytosolic tail
mIZUMO1
AA 1-21: signal peptide
AA 22-319: extracellular domain
AA 167-251: Ig-like domain
S 182
S 233
AA 204: glycosylation site
AA 320-340: transmembrane domain
AA 341-397: cytosolic tail
C
100 bp
Fw
Rv#1
Rv#2
Exon 1
Exon 9
Spaca6
gRNA#1
gRNA#2
D
Marker
wt/wt
del/wt
del/del
B6
H2O
Fw + Rv#2
Fw + Rv#1
680 bp
611 bp
E
wt/wt
gRNA#1
gRNA#2
5'-CTTCTCCTCAGACCCGGAGACCCTCTGTTATGG-3'
5'-taaagtcactgctcaccgataggtgaggacctg-3'
–8085+4
CTTCTCCTCAAAATACCTGACAAA
del/del

Figure S7. Generation of *Spaca6* gene deletion mice.

A) **Multiple alignment of SPACA6 peptide sequences from various species.** The protein sequences of bull, hamster, human, mouse, rat and zebrafish SPACA6 are aligned using the Parallel Editor of GENETYX software. Residues conserved among all displayed species are labeled in black; the ones conserved in more than half but not all of the species are highlighted in gray.
B) **Similarities between the domain structures of mouse SPACA6 and mouse IZUMO1.**
C) **Schematic presentation of *Spaca6* KO in mice using CRISPR/Cas9.** gRNAs #1 and #2 were designed to target the first coding exon and the intron downstream of the last coding exon, respectively (**SI Appendix, Table S2**). The Fw and two Rv (Rv#1 and Rv#2) primers were used to genotype the mutant mice by PCR (**SI Appendix, Table S2**).
D) **Identification of *wt* and mutant alleles by genomic PCR.** The Fw and Rv#2 primers were used to analyze the presence of the mutant allele (680 bp), whereas Fw and Rv#1 primers were used to detect the presence of the *wt* allele (611 bp).
E) **Sanger sequencing of the mutant allele.** Sanger sequencing revealed that an 8085 bp deletion and a 4 bp insertion occurred at the targeted locus of *Spaca6* in the mutant mice. Bases in uppercase and lowercase indicate exon and intron sequences, respectively.

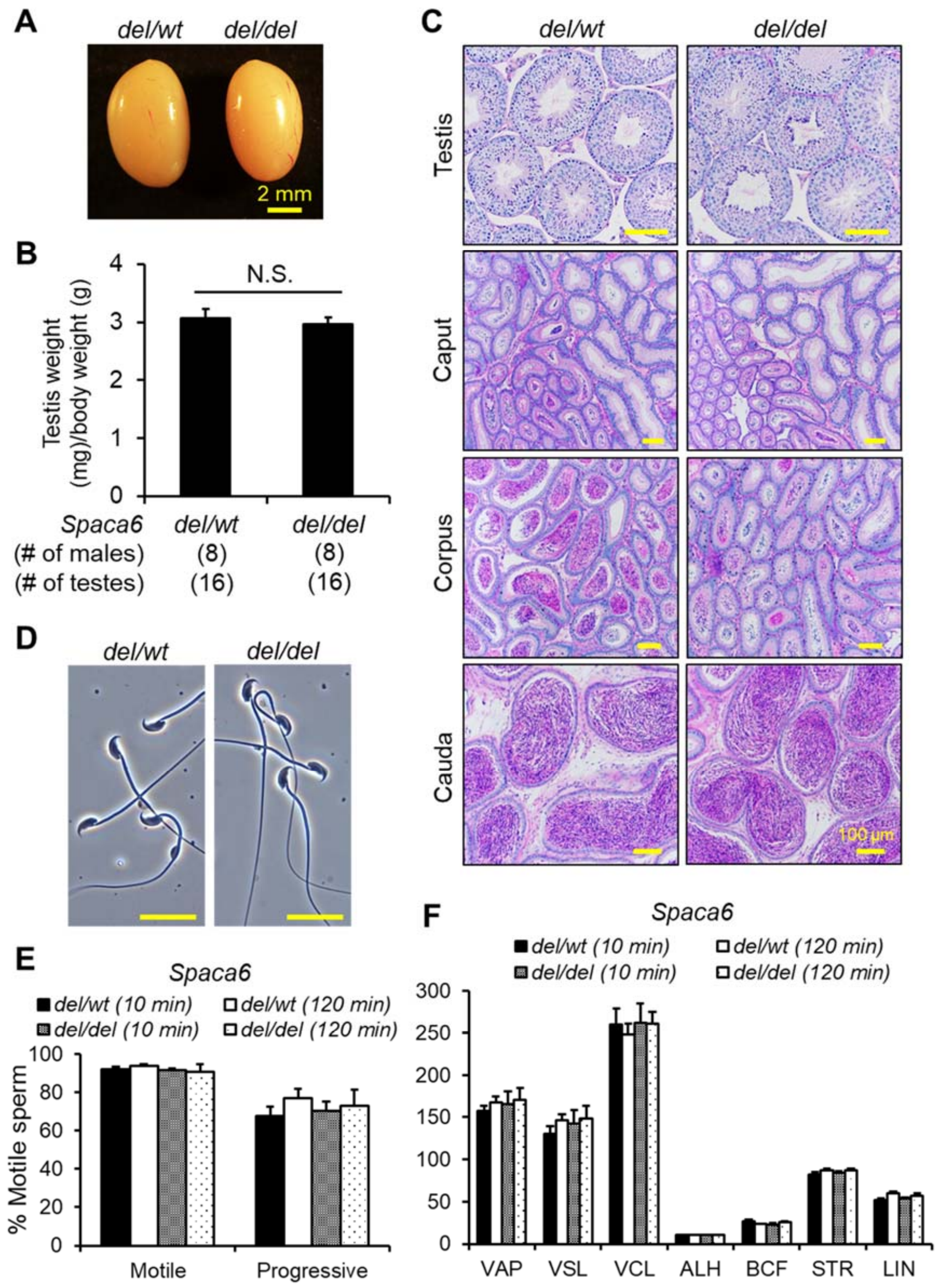
A
del/wt
del/del
2 mm
B
Testis weight (mg)/body weight (g)
N.S.
4
3
2
1
0
Spaca6
del/wt
del/del
(# of males)
(8)
(8)
(# of testes)
(16)
(16)
C
del/wt
del/del
Testis
Caput
Corpus
Cauda
100 μm
D
del/wt
del/del
E
Spaca6
del/wt (10 min)
del/wt (120 min)
del/del (10 min)
del/del (120 min)
% Motile sperm
100
80
60
40
20
0
Motile
Progressive
F
Spaca6
del/wt (10 min)
del/wt (120 min)
del/del (10 min)
del/del (120 min)
300
250
200
150
100
50
0
VAP
VSL
VCL
ALH
BCF
STR
LIN

Figure S8. Analyses of *Spaca6 del/del* testes and KO spermatozoa.

A) **Comparison of testis size and appearance in *Spaca6 del/wt* and *del/del* males.**
B) **Comparison of testis to body weight ratios in *Spaca6 del/wt* and *del/del* males.** N.S.: not significant.
C) **Histological analyses of testes and caput, corpus and cauda epididymides in *Spaca6 del/wt* and *del/del* males.** Testes and epididymides were fixed in Bouin's fluid and embedded in paraffin wax. Paraffin sections were stained by Periodic Acid Schiff and Hematoxylin.
D) **Comparison of sperm morphology in *Spaca6 del/wt* and *del/del* males.**
E) **Analyses of sperm motility and progressive movement in *Spaca6 del/wt* and *del/del* males.** Sperm motility was analyzed at 10 and 120 minutes of incubation in TYH medium using Ceros II.
F) **Analyses of sperm kinetic parameters in *Spaca6 del/wt* and *del/del* males.** Each parameter was measured at 10 and 120 minutes of incubation in TYH medium using Ceros II.

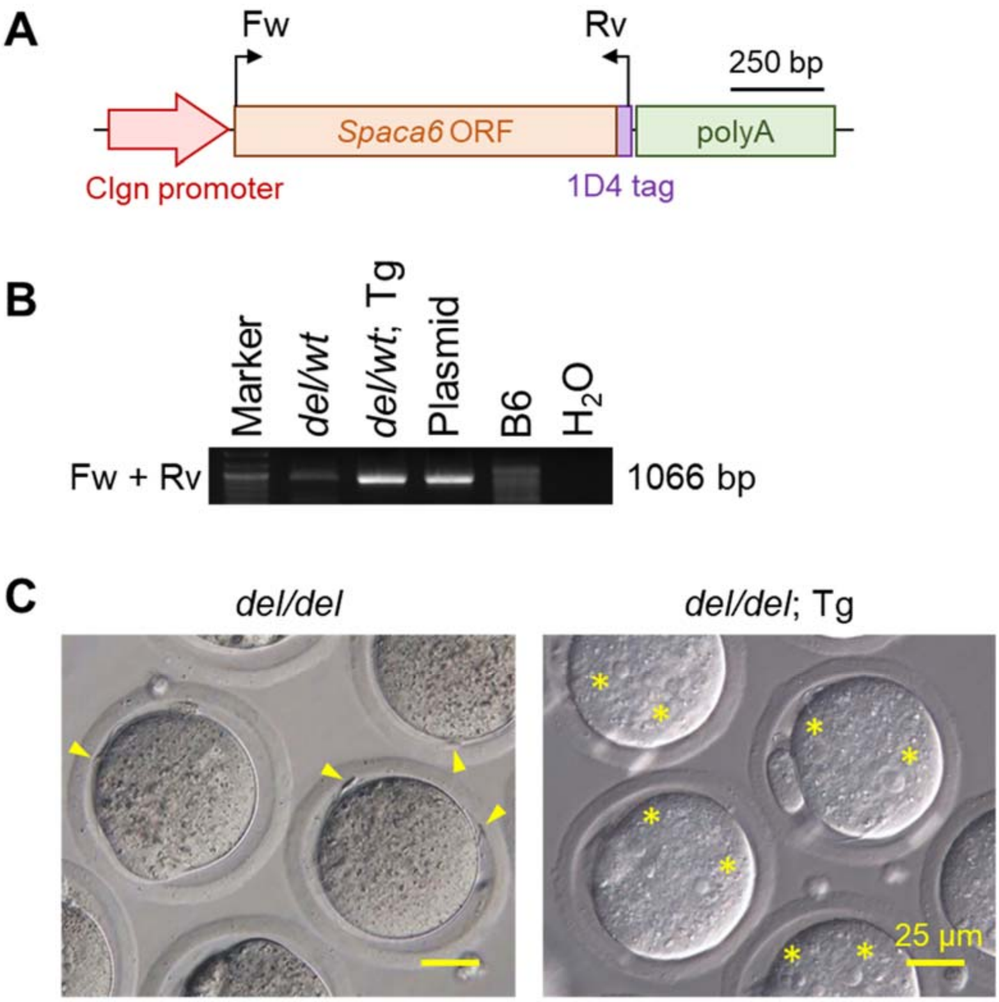

Figure S9. Generation of *Spaca6*-1D4 Tg mice.

A) **Schematic presentation of generating *Spaca6*-1D4 Tg mice by microinjection of linearized plasmid into the pronuclei of zygotes.** The expression vector contained a *Clgn* promoter upstream of the *Spaca6* ORF and polyA downstream of an 1D4 sequence. The Fw and Rv primers were designed to identify mice bearing the transgene (**SI Appendix, Table S3**).
B) **Identification of Tg mice carrying *Spaca6*-1D4 by PCR.** The Fw and Rv primers were used to analyze the presence of the transgene *Spaca6-1D4* (1066 bp). The original expression vector p*Clgn-Spaca6*-1D4 was utilized as a positive control to indicate the expected size of PCR amplicon.
C) **Analysis of the fecundity of *Spaca6* KO Tg males expressing SPACA6-1D4.** Male mice were paired with hormone-treated *wt/wt* females and oocytes were harvested from the females about 9 hours after copulation. While the Tg spermatozoa on the *Spaca6 del/del* background successfully fertilized the *wt/wt* oocytes [as indicated by the formation of 2 PN (yellow asterisks)], the *Spaca6* KO spermatozoa accumulated in the perivitelline space (yellow arrowheads) and could not fuse with oocytes.

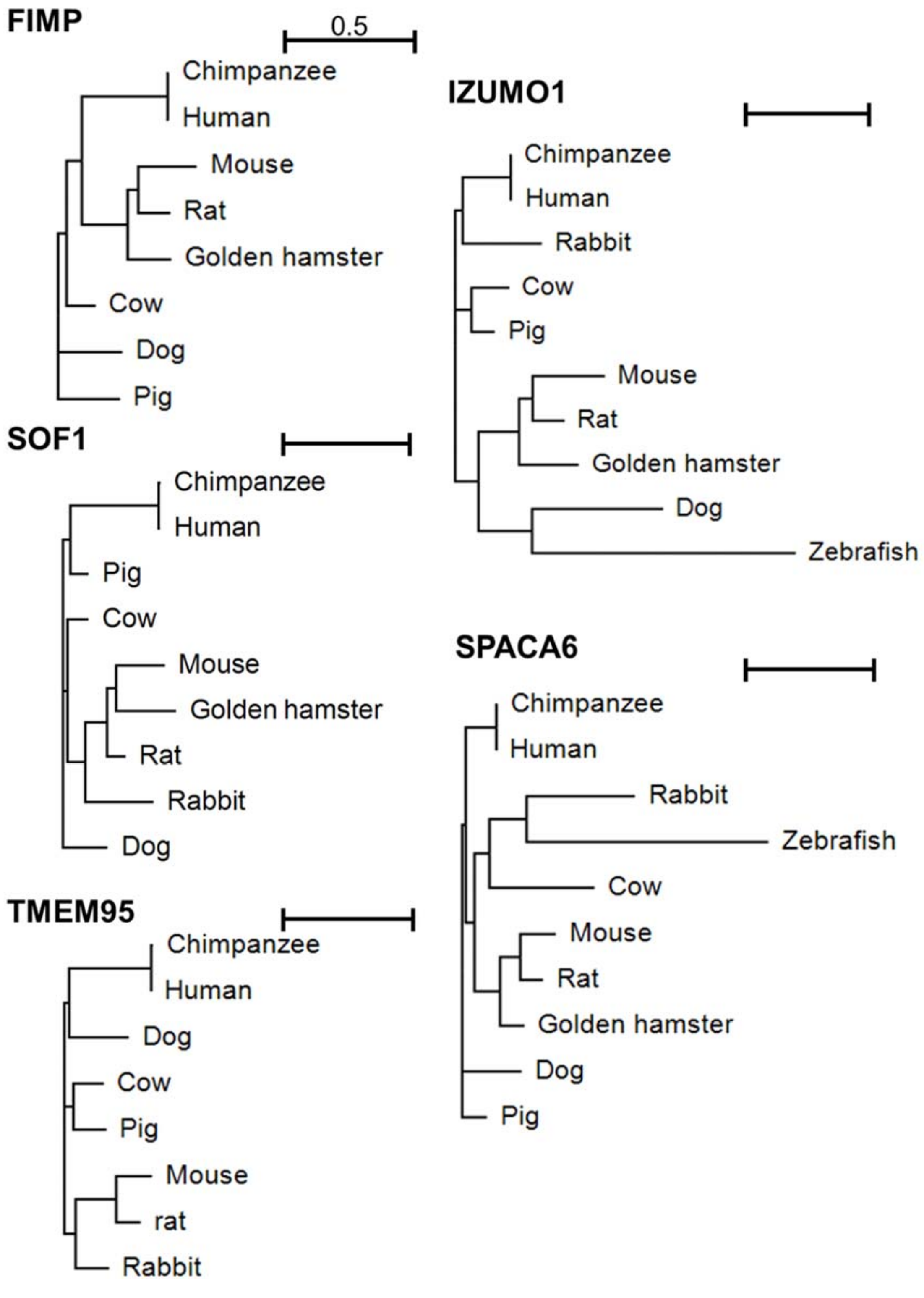

Figure S10. Phylogenic trees of FIMP, IZUMO1, SOF1, SPACA6, and TMEM95. Protein sequences of each gene in chimpanzee, cow, dog, hamster, human, mouse, pig, rabbit, rat, and zebrafish were used for the alignment.

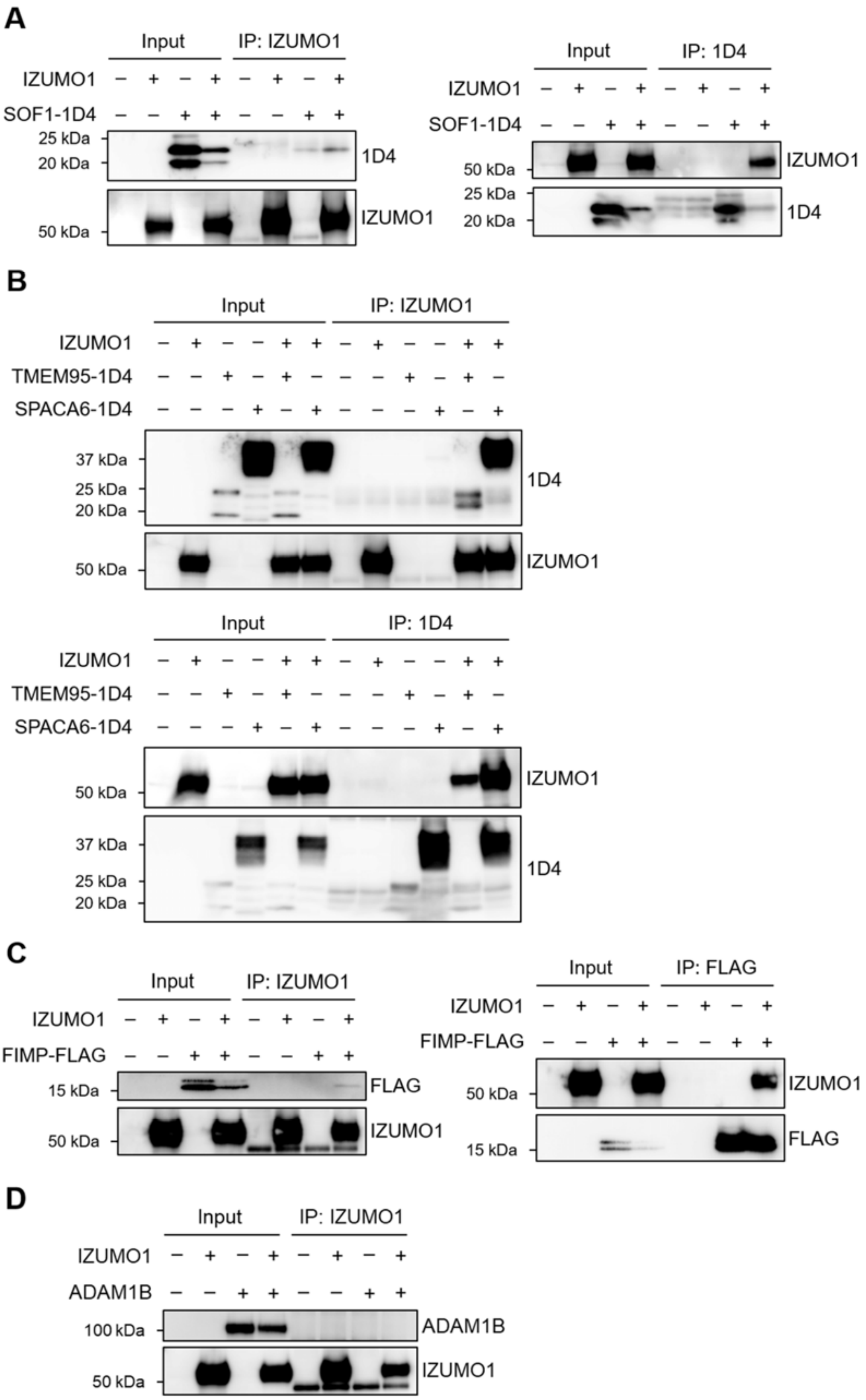
A
Input
IP: IZUMO1
IZUMO1 – + – + – + – +
SOF1-1D4 – – + + – – + +
25 kDa
20 kDa
1D4
50 kDa
IZUMO1
Input
IP: 1D4
IZUMO1 – + – + – + – +
SOF1-1D4 – – + + – – + +
50 kDa
IZUMO1
25 kDa
20 kDa
1D4
B
Input
IP: IZUMO1
IZUMO1 – + – – + + – + – – + +
TMEM95-1D4 – – + – + – – – + – + –
SPACA6-1D4 – – – + – + – – – + – +
37 kDa
25 kDa
20 kDa
1D4
50 kDa
IZUMO1
Input
IP: 1D4
IZUMO1 – + – – + + – + – – + +
TMEM95-1D4 – – + – + – – – + – + –
SPACA6-1D4 – – – + – + – – – + – +
50 kDa
IZUMO1
37 kDa
25 kDa
20 kDa
1D4
C
Input
IP: IZUMO1
IZUMO1 – + – + – + – +
FIMP-FLAG – – + + – – + +
15 kDa
FLAG
50 kDa
IZUMO1
Input
IP: FLAG
IZUMO1 – + – + – + – +
FIMP-FLAG – – + + – – + +
50 kDa
IZUMO1
15 kDa
FLAG
D
Input
IP: IZUMO1
IZUMO1 – + – + – + – +
ADAM1B – – + + – – + +
100 kDa
ADAM1B
50 kDa
IZUMO1

Figure S11. Interactions between IZUMO1 and other fusion-related proteins.

A) **Analysis of the interaction between IZUMO1 and SOF1 using HEK293T cells.** Co-IP with either anti-IZUMO1 or anti-1D4 antibody showed IZUMO1 could interact with SOF1-1D4 in HEK293T cells. SOF1-1D4 appeared as a protein doublet but only the upper band was found to interact with IZUMO1.

B) **Analysis of the interactions between IZUMO1 and TMEM95 or IZUMO1 and SPACA6 using HEK293T cells.** Co-IP with either anti-IZUMO1 or anti-1D4 antibody indicated IZUMO1 interacted with TMEM95-1D4 and SPACA6-1D4 in HEK293T cells. TMEM95-1D4 was detected as a protein triplet. Only the upper two bands but not the lower band interacted with IZUMO1.

C) **Analysis of the interaction between IZUMO1 and FIMP using HEK293T cells.** Co-IP with either anti-IZUMO1 or anti-FLAG antibody indicated IZUMO1 interacted with FIMP-FLAG in HEK293T cells. FIMP-FLAG was detected as a doublet in HEK293T cells but only the lower band interacted with IZUMO1.

D) **Analysis of the interaction between IZUMO1 and ADAM1B using HEK293T cells.** ADAM1B localizes to the sperm surface and is not involved in sperm-oocyte membrane fusion. The interaction between IZUMO1 and ADAM1B was tested as a negative control. Co-IP with anti-IZUMO1 antibody and immunoblotting with anti-ADAM1B antibody indicated IZUMO1 could not interact with ADAM1B in HEK293T cells.

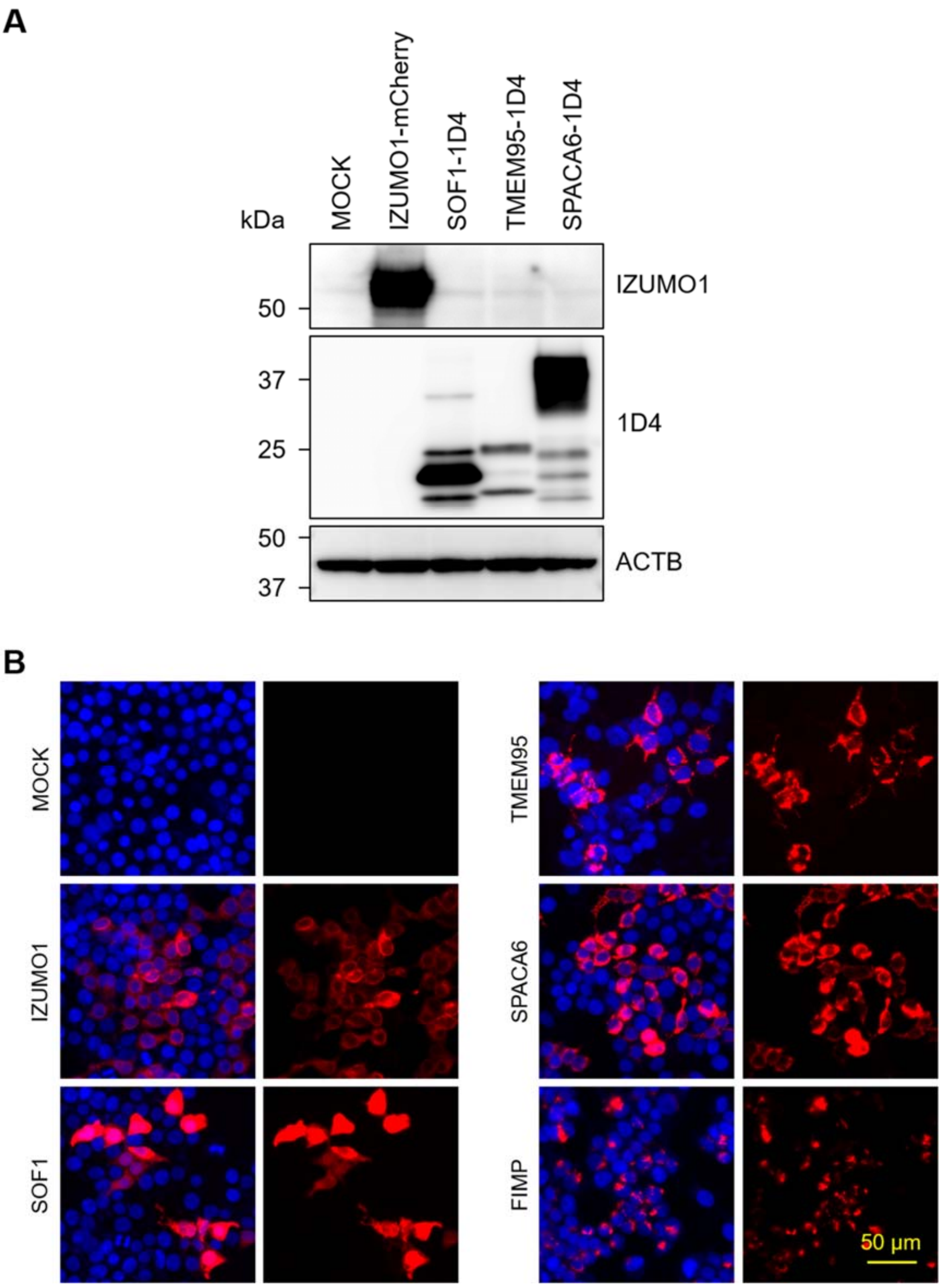
A
kDa
MOCK
IZUMO1-mCherry
SOF1-1D4
TMEM95-1D4
SPACA6-1D4
50
IZUMO1
37
25
1D4
50
37
ACTB
B
MOCK
IZUMO1
SOF1
TMEM95
SPACA6
FIMP
50 μm

Figure S12. Validation of recombinant protein expression in HEK293T cells.

A) **Western blot analyses of IZUMO1-mCherry, SOF1-1D4, TMEM95-1D4 and SPACA6-1D4 in HEK293T cells.** The amount of ACTB was examined as a loading control.
B) **Immunolocalization analyses of IZUMO1-mCherry, SOF1-1D4, TMEM95-1D4, SPACA6-1D4 and FIMP-FLAG in HEK293T cells.** HEK293T cells were stained with Hoechst 33342 and primary and Alexa Fluor 546-conjugated secondary antibodies to visualize the chromatin and recombinant proteins, respectively. All images were captured at a same exposure time.

Table S1. Primers and PCR conditions for tissue expression analyses.

Genes	Accession #	Forward (5'-3')	Reverse (5'-3')	Total cycles	Predicted size (bp)
Sof1	ENSMUST00000031900	atgacttctctgggctccca	taaaaaaaacatgatgctgg	35	324
Tmem95	ENSMUSG00000094845	gatcatgccttggcaaaccg	cgtgactgtctgggtagcag	35	531
Spaca6	ENSMUSG00000080316	gcattgtcctgttgttcctgc	ggcccgtcacgttcagataa	35	1017
Actb	ENSMUSG00000029580	catccgtaaagacctctatgccaac	atggagccaccgatccaca	30 or 35	171

Table S2. gRNAs for KO generation and primers and PCR conditions for genotyping mutant mice.

Genes	Accession #	gRNAs (5'-3')	Forward (5'-3')	Reverse (5'-3')	Total cycles	Predicted size wild/mutant (bp)
Sof1	ENSMUSG00000029867	tctgatagtgagtgcaagct (gRNA#1), agccatggcagcgatgtcat (gRNA#2), tatgagagactttgaggtgg (gRNA#3)	aagctccaatggaggttactg	cgggacatgaaattctggg	40	1544/1543 (*em1*), 520 (*em2*), 505 (*em3*)
Tmem95	ENSMUSG00000094845	attctgacccttcatgtcgg (gRNA#1), ctgtccattcccggaaagcg (gRNA#2)	gctgggtaaggaaggcctaag	agagcaacatctacaggcca	40	2119/200
Spaca6	ENSMUSG00000080316	acagagggtctccgggtctg (gRNA#1), tcactgctcaccgataggtg (gRNA#2)	ctgagctttttgaggcatctcctg	tgtcatgatcctgtacttggc/cag aaaagggtttcagctgatgcc	40	611/680

Table S3. Primers and PCR conditions for genotyping Tg mice.

Genes	Forward (5'-3')	Reverse (5'-3')	Total cycles	Predicted size (bp)
Sof1	ttgagcgggccgcttgcgcactgg	gccacaccagccaccaccttctg	40	725
Tmem95	ttgagcgggccgcttgcgcactgg	caggcgccacttggctggtc	40	838
Spaca6	atgacctcacaaaggtcact	caggcgccacttggctggtc	40	1066

Movie S1. Observation of oocytes collected from females mated with *Sof1 del/del* males (*em2*).

Movie S2. Observation of oocytes collected from females mated with *Sof1 del/del* males (*em3*).

Movie S3. Observation of oocytes after IVF using *Sof1* KO spermatozoa.

Movie S4. Observation of oocytes after IVF using *Tmem95* KO spermatozoa.

Movie S5. Observation of oocytes after IVF using *Spaca6* KO spermatozoa.

References

1. Hermann BP, *et al.*, The Mammalian Spermatogenesis Single-Cell Transcriptome, from Spermatogonial Stem Cells to Spermatids. *Cell Rep*, **25**, 1650-1667.e8, 2018.
2. Sado Y, Inoue S, Tomono Y, Omori H, Lymphocytes from enlarged iliac lymph nodes as fusion partners for the production of monoclonal antibodies after a single tail base immunization attempt. *Acta Histochem Cytochem*, **39**, 89-94, 2006.
3. Kishiro Y, Kagawa M, Naito I, Sado Y, A novel method of preparing rat-monoclonal antibody-producing hybridomas by using rat medial iliac lymph node cells. *Cell Struct Funct*, **20**, 151-156, 1995.
